# Bioengineering Platelets Presenting PD‐L1, Galectin‐9 and BTLA to Ameliorate Type 1 Diabetes

**DOI:** 10.1002/advs.202501139

**Published:** 2025-02-28

**Authors:** Yumeng Ma, Fanqiang Meng, Zhongda Lin, Yanjun Chen, Tianyu Lan, Zhaoxin Yang, Rui Diao, Xiaozhou Zhang, Qi Chen, Chi Zhang, Yishi Tian, Chanjuan Li, Wenli Fang, Xin Liang, Xudong Zhang

**Affiliations:** ^1^ Shenzhen Key Laboratory for Systems Medicine in Inflammatory Diseases School of Medicine Shenzhen Campus of Sun Yat‐Sen University Sun Yat‐Sen University Shenzhen Guangdong 518107 P. R. China; ^2^ Department of Pharmacology Molecular Cancer Research Center School of Medicine Shenzhen Campus of Sun Yat‐sen University Sun Yat‐sen University Shenzhen Guangdong 518107 P. R. China; ^3^ Guangdong Provincial Key Laboratory of Medical Molecular Diagnostics Key Laboratory of Stem Cell and Regenerative Tissue Engineering School of Basic Medical Sciences Guangdong Medical University Dongguan 523808 P. R. China; ^4^ The Affiliated Dongguan Songshan Lake Central Hospital Guangdong Medical University Dongguan Guangdong 523806 P. R. China

**Keywords:** immune checkpoints, macrophage, platelet, type 1 diabetes (T1D)

## Abstract

Autoimmune destruction of pancreatic *β*‐cells leads to impaired insulin production and onset of type 1 diabetes (T1D). Hence, immunomodulation of pancreas‐infiltrated immune cells especially the *β*‐cells autoreactive‐T cells is a promising way to hinder and reverse the progress of T1D. Herein, megakaryocytes are primed with interferon‐*γ* (IFN‐*γ*) to produce platelets presenting high levels of immunosuppressive checkpoint ligands including programmed death‐ligand 1 (PD‐L1), Programmed Death‐Ligand 2 (PD‐L2), the B and T lymphocyte attenuator (BTLA) and Galectin‐9 (Gal‐9), termed as IFN‐*γ* platelets. The IFN‐*γ* platelets bound and interacted with T cells through immune checkpoint ligands and receptors, which efficaciously induced T cell exhaustion and apoptosis in vitro. Virtually, NOD diabetes mice received IFN‐*γ* platelets treatments prominently preserved *β*‐cell integrity and insulin production, ultimately hindering the progress to hyperglycemia. Intriguingly, both the amount and activity of the pancreas infiltrate‐T cells intensively reduced, whereas the magnitude of regulatory T cells (Tregs) remarkably increased, which is attributed to IFN‐*γ* platelets treatments. Moreover, IFN‐*γ* platelets treatment instigated macrophage polarization toward an anti‐inflammatory M2 phenotype that may stimulate pancreatic angiogenesis, and promote *β*‐cell proliferation, consequently ameliorating the new‐onset T1D.

## Graphical abstract

IFN‐*γ*‐primed platelets enriched with immunosuppressive ligands (PD‐L1, PD‐L2, BTLA, and Gal‐9) are shown to induce T cell exhaustion and apoptosis, preserving *β*‐cell function and preventing hyperglycemia progression in type 1 diabetes. IFN‐*γ* platelets also promote Treg expansion, macrophage polarization, and *β*‐cell proliferation, offering a promising immunotherapy strategy for managing new‐onset type 1 diabetes.

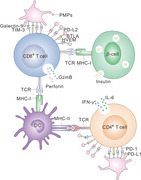

## 1 Introduction

Type 1 diabetes (T1D) is typically attributed to a combination of environmental factors, genetic predisposition, and viral infection, leading to an autoimmune response characterized by the infiltration of pancreatic lymphocytes.^[^ ^1^, ^2^, ^3^, ^4^, ^5^ ^]^ These include dendritic antigen‐presenting cells (DCs), macrophage, in particular CD4^+^ and CD8^+^ T cells.^[^ ^6^, ^7^, ^8^ ^]^ Initially, M1 macrophages promote inflammation and directly damage *β* cells, causing a gradual loss of pancreatic *β*‐cell function, which ultimately contributing to the onset and progression of the disease.^[^ ^9^ ^]^ Studies indicate that the onset of type 1 diabetes is associated with disturbances in the structure and function of the gut microbiota.^[^ ^10^ ^]^ Dysfunction or hyperactivation of DCs can result in uncontrolled autoimmune responses, representing one of the mechanisms involved in the pathogenesis of type 1 diabetes. Pancreatic islet reactive CD4^+^ and CD8^+^ T cells release cytokine such as interferon‐gamma (IFN‐*γ*), granzyme B/perforin protein particles, even exosomes to attack the *β*‐cells.^[^ ^7^, ^11^, ^12^ ^]^ Additionally, IFN‐*γ* is capable of activating type 1 macrophages and inducing the synthesis of pro‐inflammatory cytokines such as interleukin‐1*β* (IL‐1*β*) and tumor necrosis factor (TNF). These cytokines play a significant role in the degradation of *β*‐cells.^[^ ^13^, ^14^ ^]^ Hence, therapeutic strategies aimed at modulating autoreactive lymphocytes show considerable potential in addressing T1D.^[^ ^15^, ^16^, ^17^ ^]^ Notably, CD3 targeted depletion of pancreatic antigen‐specific autoreactive T cells using agents like teplizumab has been shown to enhance sustained insulin production in recently diagnosed individuals.^[^ ^18^, ^19^ ^]^ Nevertheless, concerns persist regarding the safety and effectiveness of this non‐antigen‐specific intervention. Alternatively, autologous antigen vaccines work by expanding antigen‐specific CD4^+^CD25^+^ Treg cells, which leads to either T cell deletion or anergy, thereby aiding in the prevention of type 1 diabetes onset.^[^ ^20^ ^]^ Currently, insulin injection is still the main treatment for type 1 diabetes.^[^ ^21^, ^22^ ^]^ Intriguingly, oral sustained‐release insulin nanoparticles exhibit the capacity of reduce blood glucose levels and demonstrate favorable therapeutic effects.^[^ ^23^, ^24^, ^25^ ^]^

**Scheme 1:**
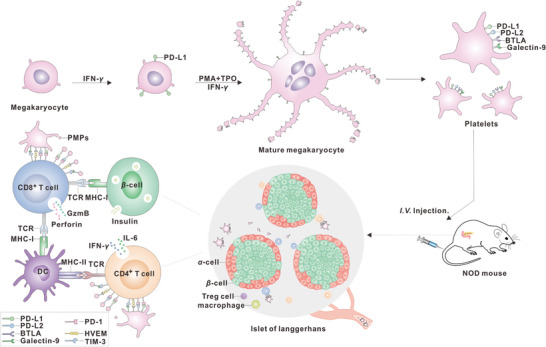
Platelets highly presenting PD‐L1, PD‐L2, BTLA and Galectin‐9 to modulate immunity in NOD diabetic mice. The mouse megakaryocytes were primed with the cytokine IFN‐*γ* to generate platelets that enrichment of PD‐L1, PD‐L2, BTLA, and Gal‐9. These modified platelets then interacted with PD‐1, HVEM, and TIM‐3 receptors on autoreactive T cells. This interaction not only inhibited the T cells activity but also induced T cell apoptosis, thereby protecting *β*‐cell from autoimmune damage.

Receptors known as T cell checkpoints, including programmed cell death protein 1 (PD‐1), T‐cell immunoglobulin and mucin‐domain containing‐3 (TIM‐3), herpesvirus entry mediator (HVEM), and CTLA‐4, are present on the surface of activated T cells. These checkpoints serve to modulate T cell activity, thereby helping to avert autoimmune responses.^[^ ^26^, ^27^ ^]^ Normal tissues express programmed death ligand 1 (PD‐L1), an important immune checkpoint ligand that prevents CD8^+^ cytotoxic T cells from triggering autoimmune responses.^[^ ^28^ ^]^ When PD‐L1 interacts with PD‐1, it leads to T cell exhaustion, which is crucial for maintaining peripheral tolerance.^[^ ^29^ ^]^ Thus, a lack of the PD‐1/PD‐L1 suppressive pathway can lead to the onset of T1D in mice.^[^ ^30^, ^31^ ^]^ Furthermore, patients with cancer treated with inhibitors targeting the PD‐1/PD‐L1 pathway are at a heightened risk of developing autoimmune diabetes, indicating a vital role for PD‐L1 in preventing the development of this disease.^[^ ^32^ ^]^ Beyond the PD‐1/PD‐L1 interaction, the signaling pathways involving B and T lymphocyte attenuator (BTLA)/HVEM and Galectin‐9/TIM‐3 are also essential in upholding peripheral tolerance.^[^ ^33^ ^]^ Therefore, immune‐suppressive checkpoint ligands such as exogenous PD‐L1, BTLA, and Galectin‐9 hold promise as therapeutic proteins for treating T1D. Significantly increasing the expression of PD‐L1 in hematopoietic stem cells, progenitor cells, human pancreatic‐like organs, platelets, and exosomes can effectively reduce autoimmune reactions and slow down the progression of T1D.^[^ ^34^, ^35^, ^36^, ^37^, ^38^, ^39^ ^]^ Alternatively, CTLA‐4 as another immune intervention target, CTLA‐4Ig is developed to block CD28 costimulatory ligands, thereby delaying diabetes onset in NOD mice.^[^ ^27^ ^]^ Significantly, using genetically altered dendritic cells (DCs) that express BTLA has been shown to induce considerable tolerance in CD8^+^ T cells and reduce the severity of diabetes in NOD mice. This approach highlights the potential of targeted immunotherapy in managing autoimmune conditions.^[^ ^40^ ^]^ Additionally, overexpression of Gal‐9 in NOD mice prolonging pancreatic islet transplant survival and preventing diabetes development by inducing apoptosis of PD‐1^+^ TIM‐3^+^ T cells in the pancreas.^[^ ^41^, ^42^ ^]^ Hence, restraining lymphocyte activity through immunosuppressive checkpoint ligands represents a promising therapeutic approach for treating autoimmune diabetes **Scheme** **1**.

Blood platelets are produced from megakaryocytes (MKs) and are essential for various physiological processes, including thrombosis, inflammation, hemostasis, immune responses, and cancer metastasis.^[^ ^43^, ^44^, ^45^, ^46^ ^]^ Consequently, platelets and platelet‐mimicking nanoparticles have been employed in developing drug delivery strategies for treating various diseases.^[^ ^47^, ^48^, ^49^, ^50^, ^51^, ^52^, ^53^ ^]^ Platelets are involved in regulating immune responses by directly interacting with a variety of immune cells, including T cells, dendritic cells, and neutrophils.^[^ ^54^, ^55^, ^56^ ^]^ Furthermore, platelets play a role in suppressing T lymphocytes, which supports anti‐inflammatory therapies for treating rheumatoid arthritis.^[^ ^57^ ^]^ Moreover, these cells secrete TGF‐*β*, a substance that suppresses the immune defense against cancer.^[^ ^58^ ^]^ Critically, existing studies indicate that age‐related *β*‐cell proliferation in pancreatic islets, influenced by signals through the platelet‐derived growth factor receptor (PDGFR) in both mice and humans, promotes *β*‐cell development and may potentially reverse diabetes.^[^ ^59^ ^]^ In this study, we stimulated megakaryocytes, platelet original cells, with cytokines IFN‐*γ* in vitro to generate substantial platelets that enrichment of immunosuppressive checkpoint ligands including PD‐L1, Programmed Death‐Ligand 2(PD‐L2), BTLA and Galectin‐9 (referred to as IFN‐*γ* platelets). IFN‐*γ* platelets were able to efficaciously restrict the activity of the *β*‐cell autoreactive T cells. IFN‐*γ* platelets could execute as immune modulator to suppress self‐reactive immune cells to hindering the progress of the new‐onset T1D.

## 2 Results

### 2.1 Generation of IFN‐*γ*‐Primed Platelets

**Figure 1:**
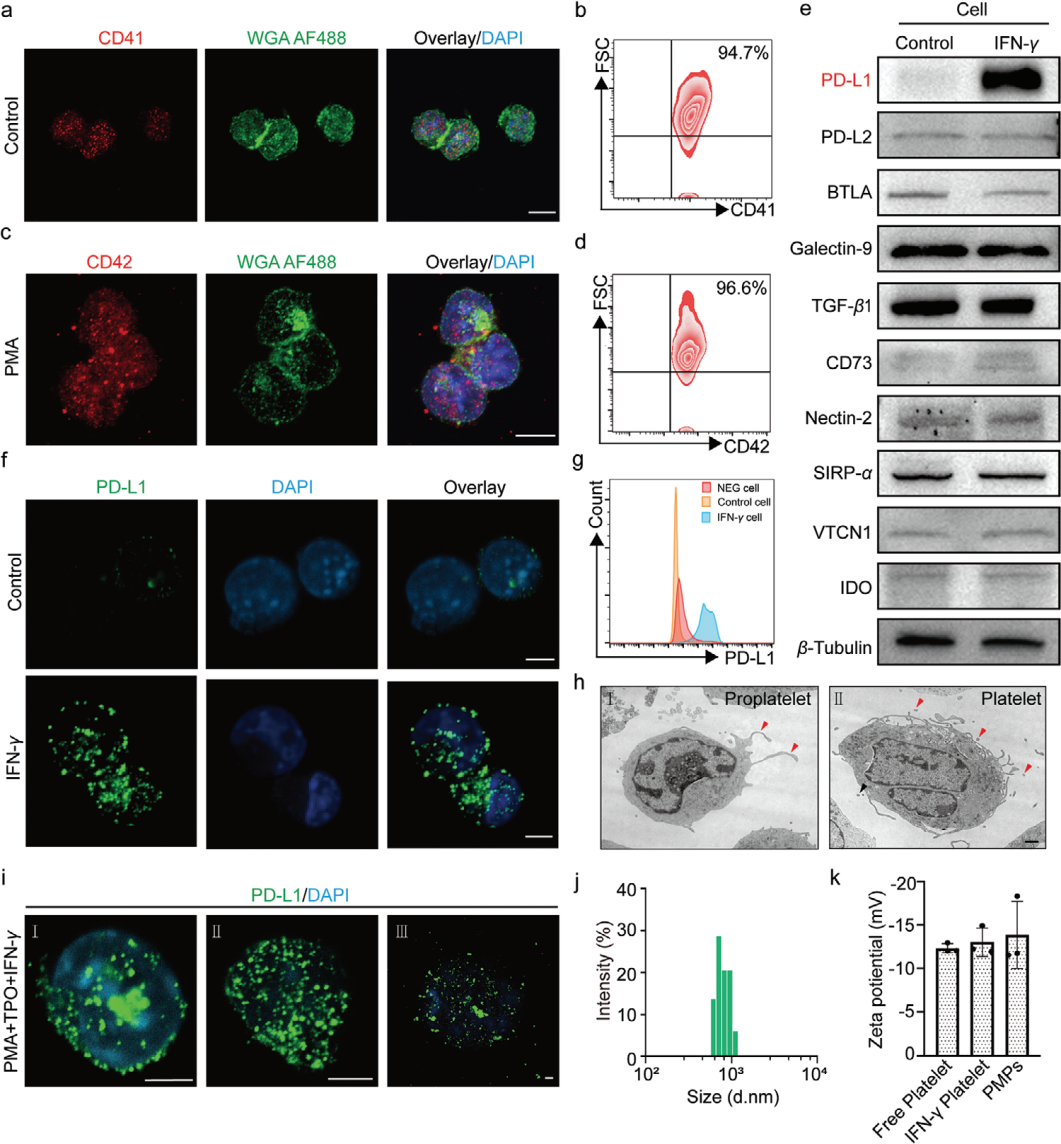
Generation of IFN‐*γ* primed platelets a,b) The mouse megakaryocytes marker CD41 expression was detected by immunofluorescence staining and flow cytometry on L8057 cells. Scale bar: 5 µm. c,d) CD42 expression on L8057 cells was detected by immunofluorescence staining and flow cytometry after 500 nM PMA treatment. Scale bar: 5 µm. e) Western blot (WB) was used to detect the expression of PD‐L1, PD‐L2, BTLA, Galectin‐9, TGF‐*β*1, CD73, Nectin‐2, SIRP‐*α*, VTCN1, and IDO before and after IFN‐*γ* stimulation in L8057 cells. f) PD‐L1 expression on L8057 cells was detected by immunofluorescence staining before and after IFN‐*γ* stimulation. Scale bar: 5 µm. g) PD‐L1 expression on L8057 cells was detected by flow cytometry before and after IFN‐*γ* stimulation. h) L8057 cells undergo different stages of maturation and differentiation were observed by transmission electron microscopy (TEM). Scale bar: 5 µm: I) the outgrowth of proplatelets; II) the release of proplatelets. i) PD‐L1 expression on L8057 cells undergo different stages of maturation and differentiation was observed by immunofluorescence staining. Scale bar: 5 µm: I) mature L8057 cells; II) the extension of pre‐platelets; III) the release of pre‐platelets. j) The size distribution of IFN‐*γ* platelets was detected by dynamic light scattering (DLS). k) Zeta potential of Free platelet, IFN‐*γ* platelet and PMPs.

To generate mouse platelets in vitro, we employed a mouse MK progenitor cell line L8057 cells. The L8057 cells underwent maturation, differentiation, and release of platelets following stimulation with phorbol 12‐myristate‐13‐acetate (PMA) and thrombopoietin (TPO) (Figure S1, Supporting Information). Polyploid nuclei were observed in mature L8057 cells (Figure S2, Supporting Information). During the initial stages of the experiment, we identified the MK cell marker CD41 in L8057 cells using flow cytometry (**Figure** **1**a,b). As an indicator of MK cell maturation, CD42 was prominently expressed in stimulated L8057 cells (Figure 1c,d). Additionally, mature L8057 cells were found to express platelet markers such as Glycoprotein VI (GPVI) and P‐selectin (Figure S3, Supporting Information). Cell surface immune checkpoint axis and metabolizing enzyme such as PD‐L1/PD‐1, PD‐L2/PD‐1, BTLA/HVEM, Galectin‐9/TIM‐3, TGF‐*β*1/TGF‐*β*RI, TGF‐*β*1/TGF‐*β*RII, CD73 (ecto‐5'‐nucleotidase), Nectin‐2/Dnam‐1, signal‐regulatory protein *α* (SIRP‐*α*)/CD47, V‐domain Ig suppressor of T cell activation (VTCN1) and Indoleamine 2,3‐Dioxygenase (IDO) play crucial roles in modulating immune cell activity and responses, thereby maintaining immune system homeostasis and preventing autoimmune diseases.^[^ ^26^ ^]^ Subsequently, experiments were performed to assess the expression of various immunosuppressive ligands and molecular, including PD‐L1, PD‐L2, BTLA, Galectin‐9, TGF‐*β*1, CD73, Nectin‐2, SIRP‐*α*, VTCN1, and IDO, in L8057 cells primed with or without IFN‐*γ* using Western blot analysis. The results revealed a significant upregulation of PD‐L1 following IFN‐*γ* stimulation, while no significant changes were detected in the expression levels of the other proteins (Figure 1e). The enhanced expression of PD‐L1 in L8057 cells after IFN‐*γ* stimulation was further confirmed through laser confocal microscopy and flow cytometry (Figure 1f,g). Whereas the expression of PD‐L2, BTLA, and Galectin‐9 in L8057 cells were almost no change treated with or without IFN‐*γ* by laser confocal microscopy (Figure S4, Supporting Information). Furthermore, we observed the generation and release of proplatelets during the maturation and differentiation process of L8057 cells using transmission electron microscopy. This observation included witnessing proplatelet formation, where proplatelets bud off from the cell membrane and gradually detach (Figure 1h). Subsequent examination under laser confocal microscopy revealed an accumulation of PD‐L1‐rich vesicles in the cytoplasm of mature L8057 cells upon PMA stimulation, which ultimately led to platelet formation (Figure 1i). Subsequently, platelets released into the culture medium were successfully isolated via differential centrifugation. The average diameter was measured using dynamic light scattering (DLS) technology, which revealed a size range of 0.6 to 1.3 microns. Furthermore, analysis of charge characteristics was conducted on IFN‐*γ* platelets, Free platelets and PMPs using the Malvern Zetasizer Nano‐ZS to gain insights into their stability and functional properties in hemodynamics (Figure 1j,k).

### 2.2 In Vitro Biological Characteristics of Platelets

**Figure 2:**
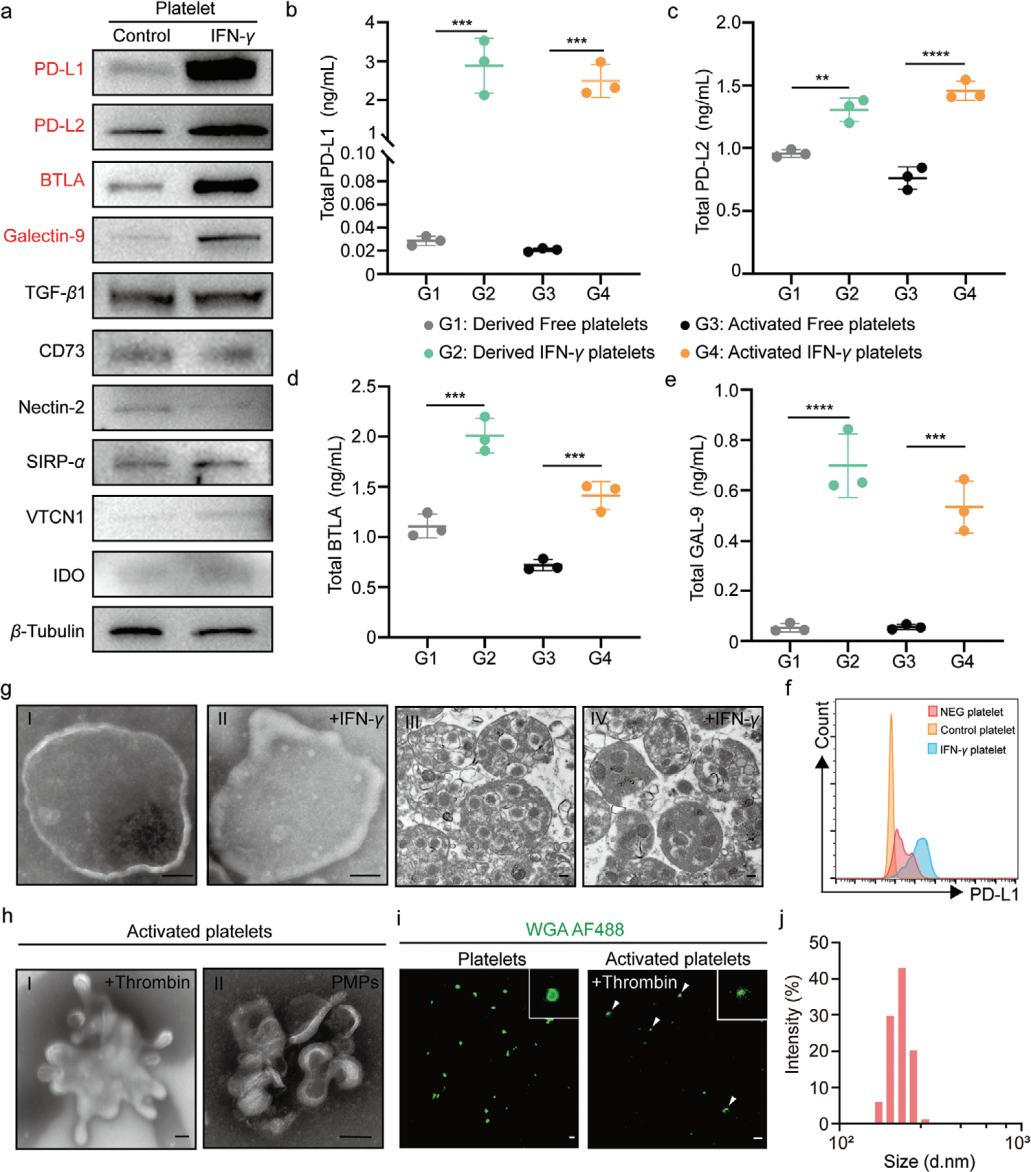
Characteristics and activation of ex vivo platelet. a) WB was used to analyze the expression levels of PD‐L1, PD‐L2, BTLA, Galectin‐9, TGF‐*β*1, CD73, Nectin‐2, SIRP‐*α*, VTCN1, and IDO before and after IFN‐*γ* stimulation of platelets to determine the changes in expression levels. b) The expression level of PD‐L1 in 1 mg of platelets was detected by enzyme‐linked immunosorbent assay (ELISA). (*n* = 3). c) The expression level of PD‐L2 in 1 mg of platelets was detected by ELISA. (*n* = 3). d) The expression level of BTLA in 1 mg of platelets was detected by ELISA (*n* = 3). e) The expression level of Galectin‐9 in 1 mg of platelets was detected by ELISA (*n* = 3). f) Flow cytometry was used to detect the expression of PD‐L1 in platelets before and after IFN‐*γ* stimulation. g) Transmission electron microscopy images of Free platelets, IFN‐*γ* platelets, and cut‐open platelets were obtained using biological transmission electron microscopy. Scale bar: 1000 nm. h) Transmission electron microscopy images of activated IFN‐*γ* platelets and released platelet microparticles (PMPs) are presented. Scale bar: 1000 nm. i) Laser confocal microscopy was used to observe unactivated platelets and activated platelets. Scale bar: 5000 nm. j) DLS was used to detect the size distribution of PMPs. Throughout the experiment, NS indicates no significant difference, *p < 0.05, **p < 0.01, ***p < 0.001; a two‐way ANOVA was performed, followed by post hoc tests for analysis.

Subsequently, we employed immunoblotting to comprehensively investigate the impact of IFN‐*γ* on the expression of ligands associated with T cell immune suppression in platelets released from L8057 cells. Our findings demonstrated a significant upregulation of PD‐L1, PD‐L2, BTLA, and Galectin‐9 in platelets following exposure to IFN‐*γ* (**Figure** **2**a). Meanwhile, we observed that the expression levels of TGF‐*β*1, CD73, SIRP‐*α*, VTCN1, and IDO remained unchanged, with no significant alterations evident (Figure 2a). Subsequently, we collected platelets from megakaryocytes stimulated with PMA and IFN‐γ for 72 h and performed an enzyme‐linked immunosorbent assay (ELISA) to further confirm that, under IFN‐*γ* stimulation, the expression of immune checkpoint inhibitory ligands, such as PD‐L1, PD‐L2, BTLA, and Galectin‐9, increased in platelets (Figure 2b–e). Hereafter, we conducted a thorough analysis of PD‐L1 expression in platelets and their progenitor cells, both before and after stimulation with IFN‐*γ*, utilizing flow cytometry (Figure 2f). Moreover, platelet‐derived TGF‐*β* exerts an inhibitory effect on the host's immune response. We also observed that TGF‐*β*1 is released from platelets, including those expressing PD‐L1, which may have implications for the treatment of T1D (Figure S5, Supporting Information). Our research findings demonstrated that the elevated level of PD‐L1 on IFN‐*γ*‐primed platelets resulted from the induction of PD‐L1 expression in L8057 cells by IFN‐*γ*. Alternatively, the high level of PD‐L2, BTLA, and Galectin‐9 on IFN‐*γ* primed may because of the increase of protein transfer to platelets from MK cells.

Afterwards, we observed a distinct double‐layered vesicle structure in platelets, and IFN‐*γ* stimulation did not alter the content of platelet granules by transmission electron microscopy (TEM) (Figure 2g,h). Additionally, we documented the activation of the platelets and platelet microparticles (PMPs) release process, which are essential for blood clotting, thrombosis formation, inflammatory responses, and tissue repair and regeneration. TEM images further confirm the structure of the platelets and PMPs, we stimulated platelet activation in vitro and carefully documented the PMP release process (Figure 2g,h). We then used laser confocal microscopy to observe how platelets form vesicle structures and release PMP through protrusion extension during activation (Figure 2i). Additionally, we stimulated platelets with thrombin and incubated them at 37 °C for 12 h, documenting protrusion formation and PMP release by activated platelets (Figure 2i). Dynamic light scattering (DLS) analysis provided key data on the physical and functional characteristics of released PMP, including an average diameter of 0.1 to 0.4（ecto‐5'‐nucleotidase) microns, enhancing our understanding of platelet behavior (Figure 2j).

### 2.3 Comparison of the Proteomics of Free‐Platelet and IFN‐*γ*‐Platelet

**Figure 3:**
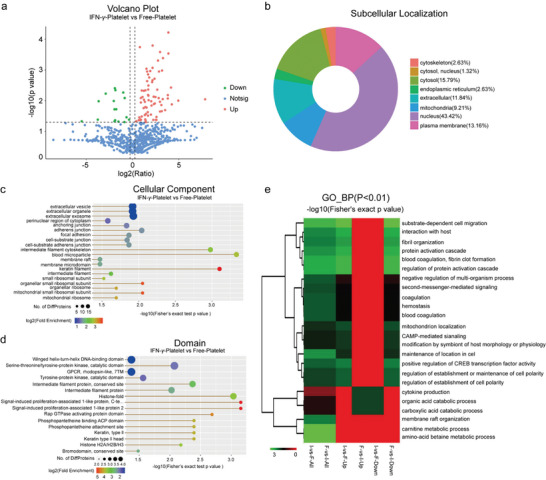
Proteomic analysis of Free‐Platelet and IFN‐*γ*‐Platelet. a) Volcano plot of differentially expressed proteins between Free‐Platelet and IFN‐*γ*‐Platelet. b) Subcellular localization of differentially expressed proteins between Free‐Platelet and IFN‐*γ*‐Platelet. c) Cellular components enriched by differentially expressed protein pathway enrichment analysis. d) Domains enriched by differentially expressed protein pathway enrichment analysis. e) Functional clustering of differentially expressed proteins based on Biological process.

We used high‐performance liquid chromatography (HPLC) to separate the proteins from Free‐platelet and IFN‐*γ*‐platelet, and then analyzed them with mass spectrometry (MS). We identified 3017 proteins and then compared the protein profiles of Free‐platelet and IFN‐*γ*‐platelet using unlabeled quantification methods. This analysis revealed 90 proteins with statistically significant differences (ratio > 1.2, *P* < 0.05). Of these, 75 proteins were upregulated in IFN‐*γ*‐platelet compared to Free‐platelets, while 17 were downregulated (**Figure** **3**a). Subsequently, we then performed gene ontology (GO) annotation on the differentially expressed proteins. GO annotation classifies proteins according to their roles in biological processes, cellular components, and molecular functions. Proteins were categorized based on their enrichment in either cellular processes and specific biological activities or cellular components and organelles (Figure S6, Supporting Information). The differentially expressed proteins showed significant variations in molecular functions between IFN‐*γ*‐platelet and Free‐platelets. Subcellular localization analysis showed that these proteins were mainly distributed in the nucleus (43.42%), cytoplasm (15.79%), and plasma membrane (13.16%) (Figure 3b). Subsequently, the differentially expressed proteins between IFN‐*γ*‐platelet and Free‐platelets were subjected to enrichment analysis using oxidized graphene oxide, along with KEGG pathway analysis for gene and genome data. The KEGG signaling pathways also revealed functional differences among the differentially expressed proteins in the IFN‐*γ*‐platelet compared to the Free‐platelet. Notably upregulated signaling pathways in IFN‐*γ*‐platelet included alcoholism, systemic lupus erythematosus, Salmonella infection, and olfactory signal transduction; conversely, downregulated pathways were observed in Free‐platelets (Figure S7, Supporting Information). Cellular components include a variety of subcellular structures, such as protein complexes, as well as other functional regions within cells. Differential expression analysis was used for pathway enrichment to identify cellular components associated with IFN‐*γ*‐platelet compared to Free‐platelets. Notably, most of the overexpressed proteins following IFN‐*γ* stimulation are predominantly enriched in blood platelet granules and keratin filaments. In contrast, proteins associated with membrane rafts and microdomains are elevated under unstimulated conditions (Figure 3c; Figure S8, Supporting Information). Moreover, the domains of overexpressed proteins post IFN‐*γ* stimulation primarily relate to conserved sites found on intermediate filament/histone folding/proliferation‐associated signaling molecules like SIPA1/SIPA2 potentially influencing immune cell growth/regulation functions. Conversely, in unstimulated conditions, the upregulation is seen for immunoglobulin‐like structural domain‐related sequences impacting immune response regulation/cell growth/metabolic processes thereby influencing overall immunity/cellular homeostasis (Figure 3d; Figure S9, Supporting Information). In contrast, the upregulated proteins in IFN‐*γ*‐platelet were predominantly enriched in matrix‐dependent cell migration, protein activation cascades, host interactions, regulation of protein activation cascades, blood coagulation, and fibrin clot formation (Figure 3e; Figure S10, Supporting Information).

### 2.4 Biological Behavior of IFN‐*γ* Platelet

**Figure 4:**
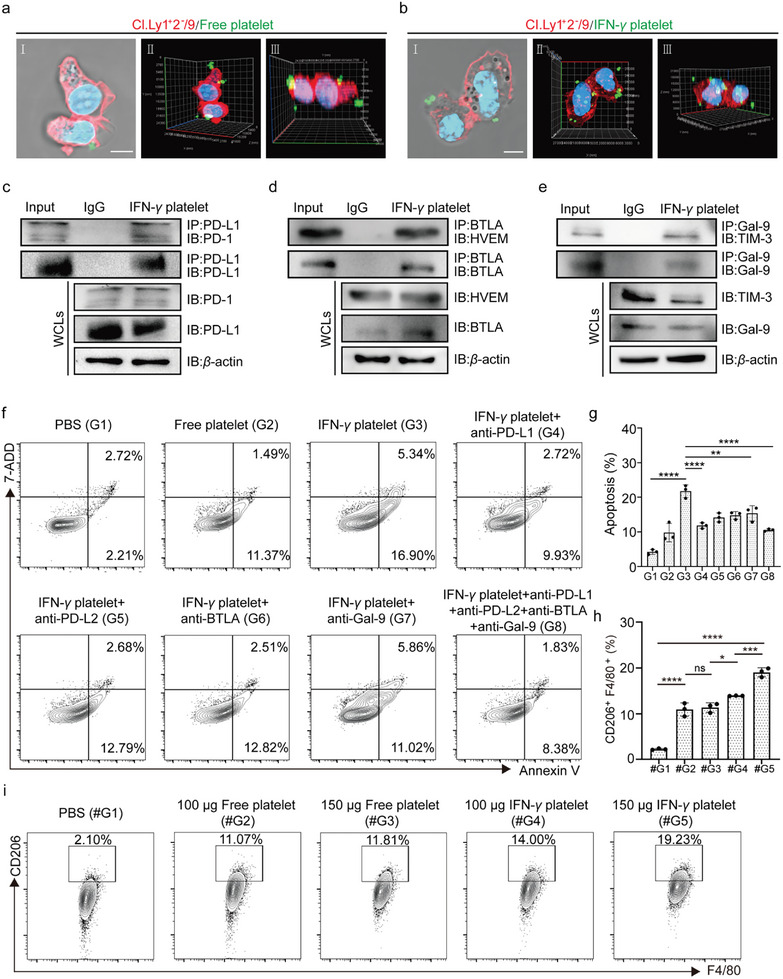
IFN‐γ Platelets induced T cell apoptosis and macrophage activation. a) 3D confocal microscopy images of CI.Ly1^+^2^−^/9 cells co‐cultured with Free platelet for 6 h observed by laser scanning confocal microscopy. b) 3D confocal microscopy images of CI.Ly1^+^2^−^/9 cells co‐cultured with IFN‐*γ* platelet for 6 h observed by laser scanning confocal microscopy. c–e) Co‐immunoprecipitation assay to investigate the interaction between PD‐L1, BTLA and Gal‐9 on IFN‐*γ* platelets and PD‐1, HVEM and TIM‐3 on CI.Ly1^+^2^−^/9 cells. f,g) Flow cytometry analysis showed representative plots (f) and statistical data (g) of cell apoptosis in different treatment groups after co‐culturing platelets and CI.Ly1^+^2^−^/9 cells for 24 h (*n* = 3). h,i) Representative flow‐cytometry data (i) and statistical data (h) to show CD206^+^ F4/80^+^ macrophage cells of cell polarization across different treatment groups after 24 h of co‐culturing with unpolarized RAW264.7 cells (*n* = 3). Throughout the experiment, NS indicates no significant difference, *p < 0.05, **p < 0.01, ***p < 0.001; a two‐way ANOVA was performed, followed by post‐hoc tests for analysis.

Afterward, we examine changes in the quantity of Free platelets and IFN**‐*γ*** platelet binding to a mouse CD4 ^+^ T cells, CI.Ly1^+^2^−^/9 cells, by laser scanning confocal microscopy (**Figure** **4**a,b). The findings showed that IFN**‐*γ*** platelets bound significantly more to CI.Ly1^+^2^−^/9 cells than Free platelets, suggesting that IFN**‐*γ*** treatment might enhance the immune regulatory functions of platelets. Based on these observations, we performed in vitro immunoprecipitation (CO‐IP) and cell‐binding assays to explore the interaction between IFN**‐*γ*** platelets and T cells. CO‐IP data showed that the PD‐L1 antibody effectively precipitated PD‐1 and PD‐L1 proteins, whereas the PD‐L2 antibody efficiently pulled down PD‐1 and PD‐L2 proteins (Figure 4c; Figure S11, Supporting Information). Additionally, the BTLA antibody effectively co‐immunoprecipitated both BTLA and HVEM proteins, while the Galectin‐9 antibody successfully co‐immunoprecipitated TIM‐3 and Gal‐9 proteins (Figure 4d,e). Hereafter, we performed antibody blockade studies to assess how these interactions affect T cell functionality. The findings revealed that blocking BTLA, GAL‐9, and PD‐L2 antibodies slightly reduced IFN**‐*γ*** platelet function, while inhibiting anti‐PD‐L1 antibodies effectively countered the suppressive effect of high PD‐L1 platelets on T cells (Figure 4f,g). Considering that platelets are rich in molecules such as TGF‐β, this study plans to continue experiments to investigate whether platelets activated by IFN‐*γ* have the ability to induce the formation of M2 macrophages.^[^ ^60^ ^]^ In subsequent experiments, we co‐cultured engineered platelets with undifferentiated RAW264.7 macrophage cells to examine the impact of their interaction on macrophage polarization. The results demonstrated that Free platelets promoted the polarization of M0 macrophages into M2 macrophages (Figure 4h,i). In comparison, IFN‐*γ* platelets led to a higher proportion of M0 macrophages polarizing to M2 macrophages (Figure 4h,i).

### 2.5 IFN‐*γ* Platelet Reverses Hyperglycemia in NOD Diabetic Mice

**Figure 5:**
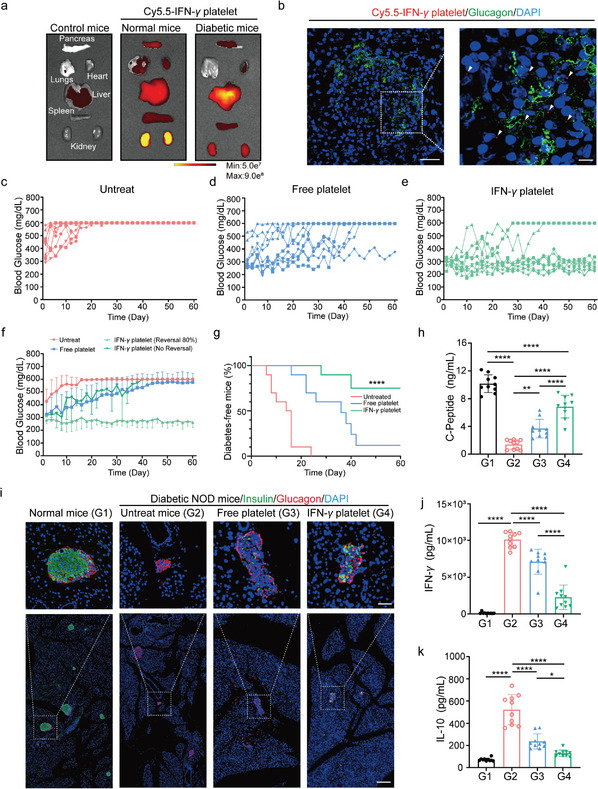
IFN‐*γ* platelets reverse hyperglycemia in NOD diabetic mice. a) Fluorescence spectral images taken after tail‐vail injection of Cy5.5‐IFN‐*γ* platelet, showing the biological distribution in major organs and pancreas. b) Pancreatic sections of diabetic NOD mice stained with Cy5.5‐IFN‐*γ* platelet. (Scale bar: 50 µm in inserts; 10 µm in enlarged images). c–e) Different treatment regimens for diabetic NOD mice, as indicated (*n* = 10). f) Average blood glucose levels in diabetic NOD mice under different treatment regimens, as indicated (*n* = 10). The dark green line represents diabetic NOD mice that were not reversed (*n* = 2); the light green line represents reversed diabetic NOD mice (*n* = 8). Data are presented as mean ± standard deviation (s.d.). g) The occurrence of NOD mice developed diabetes (*n* = 10). h) C‐Peptide concentration in serum measured by ELISA at 60 days (*n* = 10) error bars, G1: Normal mice; G2: Untreat mice; G3: Free platelet; G4: IFN‐*γ* platelet, mean ± s.d. i) Representative confocal image of insulin^+^ *β* cells in pancreatic islets. G1: Normal mice; G2: Untreat mice; G3: Free platelet; G4: IFN‐*γ* platelet (scale bar: 200 µm in inserts; 50 µm in enlarged images.). j) C‐Peptide concentration in serum measured by ELISA at 60 days (*n* = 10) error bars, G1: Normal mice; G2: Untreat mice; G3: Free platelet; G4: IFN‐*γ* platelet, mean ± s.d. k) IL‐10 concentration in serum measured by ELISA at 60 days (*n* = 10) error bars, G1: Normal mice; G2: Untreat mice; G3: Free platelet; G4: IFN‐*γ* platelet, mean ± s.d. NS indicates no significant difference; *p < 0.05, **p < 0.01, ***p < 0.001; a two‐way ANOVA was performed, followed by post hoc tests for analysis.

Initially, we conducted a comparative analysis of the fluorescence signals from platelets in both normal and T1D mice to evaluate their localization within the pancreas. The data strongly indicate that engineered platelets effectively targeted inflamed islets and generated robust fluorescence signals for in vivo imaging (**Figure** **5**a). Next, we investigated the in vivo distribution of Free platelets and IFN‐*γ* platelets in NOD mice with high blood glucose levels. Notably, both IFN‐*γ* platelets and Free platelets were identified in the pancreas of diabetic NOD mice (Figures S12–S14, Supporting Information). Additionally, the accumulation of IFN‐*γ* platelets in the pancreas of diabetic NOD mice was significantly greater than that observed in normal mice, suggesting that platelets have a natural ability to target inflamed tissues. PMPs were significantly smaller than resting platelets, which may facilitate the infiltration of IFN‐*γ* platelets into the pancreas and their interaction with T cells.^[^ ^61^ ^]^ To examine whether IFN‐*γ* platelets could induce the release of PMPs in diabetic NOD mice, we administered IFN‐*γ* platelets to these mice. Virtually, our findings show that IFN‐*γ* platelets can generate PMPs in vivo (Figure S15, Supporting Information). Most platelets were observed as single cells, indicating a low likelihood of IFN‐*γ* platelet‐induced thrombosis (Figure S15, Supporting Information). Importantly, Cy5.5‐labeled platelets were injected intravenously into NOD mice with high blood glucose levels, showing that IFN‐*γ* platelets can effectively target the pancreatic islets (Figure 5b).

To evaluate the potential of IFN‐*γ* platelets in reversing newly‐developed T1D, female NOD/ShiLtJI mice were divided into three groups: untreated, Free platelets, and IFN‐*γ* platelets. Beginning at 10 weeks of age, blood glucose levels were monitored every other day. In healthy mice, the normal blood glucose range is between 80 and 130 mg/dL^−1^. NOD mice were classified as having new‐onset diabetes when their blood glucose levels surpassed 250 mg/dL^−1^. Subsequently, Free platelets or IFN‐*γ* platelets were administered via intravenous injection every other day until the end of the 60‐day experiment. The blood glucose levels of newly diagnosed T1D mice (blood glucose > 250 mg d^−1^ L^−1^) progressively increased, eventually reaching severely high levels (> 600 mg d^−1^ L^−1^) in the untreated group. In contrast, treatment with IFN‐*γ* platelets prominently prevented T1D progression in mice and restored high blood glucose levels to normal. However, T1D progression was only partially suppressed in mice treated with Free platelets therapy, and high blood glucose levels could not be reversed (Figure 5c–f). Afterwards, we measured blood C‐Peptide levels in NOD mice. After IFN‐*γ* platelet treatment, these levels showed a threefold increase compared to untreated NOD mice (Figure 5h). Notably, treatment with IFN‐*γ* platelets effectively reversed T1D in NOD mice compared to treatment with Free platelets (Figure 5g).

Afterward, we explored the potential of engineered platelets to safeguard the remaining pancreatic *β*‐cells by limiting the activity of autoreactive T cells. We collected pancreatic tissue samples from NOD mice treated with various regimens to analyze the insulin‐secreting *β*‐cells by immunofluorescence and immunohistochemistry. In healthy mice, the insulin‐producing *β*‐cells within the pancreatic islets were preserved, encircled by *α*‐cells that secrete glucagon. Conversely, NOD mice that were not treated and had elevated blood glucose levels showed a substantial reduction in the insulin‐producing *β*‐cells within the pancreatic islets, suggesting possible destruction.″Meanwhile, *α*‐cells that secrete glucagon were found dispersed throughout the pancreas. Notably, with the IFN‐*γ* platelets treatment, insulin expression in the islets was higher compared to the untreated and Free platelets treatment mice (Figure 5i). Subsequently, we assessed pro‐inflammatory cytokine levels to determine if IFN‐*γ* platelets could reduce inflammation in NOD mice. Serum levels of IFN‐*γ* were significantly lower in mice treated with IFN‐*γ* platelets, and these platelets were more effective at reducing IFN‐*γ* than Free platelets (Figure 5j). Treg cells and M2 macrophages help regulate the immune response by producing anti‐inflammatory cytokines such as IL‐10 and TGF‐*β*.^[^ ^62^, ^63^ ^]^ As a result, we evaluated the levels of these cytokines, focusing on IL‐10, known for its role in protecting the host by damping immune reactions to pathogens and preventing tissue damage. Our findings indicated that IL‐10 levels were decreased in mice treated with platelets, suggesting a reduced inflammatory state (Figure 5k).

### 2.6 The Impact of IFN‐*γ* Platelets on the Activity of Immune Cells Infiltrating the Pancreas

**Figure 6:**
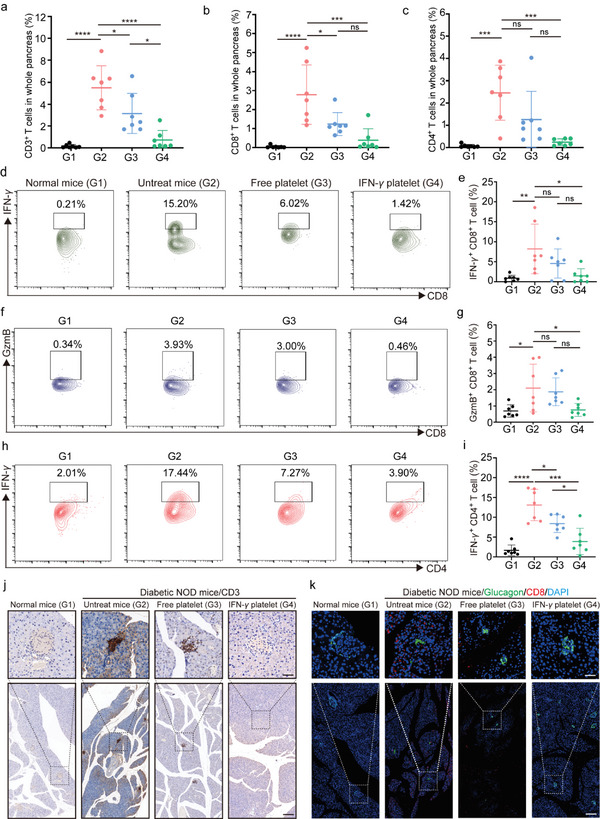
Impact of IFN‐*γ* platelets on the activity of pancreatic infiltrating T cells. a–c) Percentage of CD3^+^ (a), CD8^+^ (b), and CD4^+^ (c) T cells in total cells, G1: Normal mice; G2: Untreat mice; G3: Free platelet; G4: IFN‐*γ* platelet. d,e) Representative flow cytometry (using CD8^+^ T cell gate) analysis and quantification of the number of infected pancreatic infiltrating CD8^+^ T cells expressing IFN‐*γ* in different treatment groups (d) and statistical data (e) (*n* = 7), G1: Normal mice; G2: Untreat mice; G3: Free platelet; G4: IFN‐*γ* platelet. f,g) Representative flow cytometry (using CD8^+^ T cell gate) analysis and quantification of the number of infected pancreatic infiltrating GzmB^+^ CD8^+^ T cells in different treatment groups (f) and statistical data (g) (*n* = 7), G1: Normal mice; G2: Untreat mice; G3: Free platelet; G4: IFN‐*γ* platelet. h,i) Representative flow cytometry (using CD4^+^ T cell gate) analysis and quantification of the number of infected pancreatic infiltrating CD4^+^ T cells expressing IFN‐*γ* in different treatment groups (h) and statistical data (i) (*n* = 7), G1: Normal mice; G2: Untreat mice; G3: Free platelet; G4: IFN‐*γ* platelet. j) Immunohistochemical analysis of CD3^+^ T cells in normal mice and treated pancreatic tissues (scale bar: 200 µm in inserts; 50 µm in enlarged images.). k) Immunofluorescence analysis of CD8^+^ T cells in normal mice and treated pancreatic tissues (scale bar: 200 µm in inserts; 50 µm in enlarged images.). Throughout the experiment, NS denotes no significant difference, *p < 0.05, **p < 0.01, ***p < 0.001; a two‐way ANOVA was performed, followed by post‐hoc tests for analysis.

To develop immunomodulation strategies for type 1 diabetes, significant attention has been directed toward examining the role that T lymphocytes play in the autoimmune destruction of pancreatic *β*‐cells. Thus, we further evaluate the efficacy of IFN‐*γ* platelets in suppressing autoimmune attacks by using flow cytometry to detect CD3^+^, CD4^+^, and CD8^+^ effector T cells in the pancreas of NOD mice. Remarkably, IFN‐*γ* platelets intensively reduced the overall population of CD3^+^, CD4^+^, and CD8^+^ T cells in the pancreas in vivo (**Figure** **6**a–c). However, a notable increase of infiltration of CD3^+^, CD8^+^, and CD4^+^ T cells was observed in the pancreases of hyperglycemic mice, indicating a robust immune response. Flow cytometry analysis revealed prominent reduction in pancreatic infiltration by CD3^+^, CD8^+^, and CD4^+^ T cells after treatment with both Free platelets and IFN‐*γ* platelets. Additionally, we assessed activated CD8^+^ T cell subsets secreting cytotoxic factors, including granzyme B and IFN‐*γ*, within pancreatic tissue. Subsequently observed reductions were noted for both populations expressing these markers post‐treatment (Figure 6d–g). Notably, similar inhibitory effects on pancreatic infiltration were observed for CD4^+^ T cells when exposed to IFN‐*γ* platelets, which concurrently led to a reduced proportion of granzyme B‐expressing subsets in the treated animals compared to controls (Figure 6d–g). We hypothesized that after interacting with IFN‐*γ* platelets, infiltrating self‐reactive CD8^+^ and CD4^+^ T cells might become inactivated, thereby reducing their toxic attack on *β*‐cells. To assess the extent of pancreatic infiltration by T cells, we harvested pancreases from different treatment cohorts of NOD mice. Subsequently, we employed immunohistochemistry and immunofluorescence staining for analysis. The results of immunofluorescence staining revealed that normal mice exhibited minimal T cell infiltration in the pancreas, whereas high‐glucose NOD mice showed pronounced T cell infiltration within their islets (Figure 6j,k; Figures S16 and S17, Supporting Information). Following treatment with IFN‐*γ* platelets, an intensive reduction in pancreatic T cell infiltration was observed. In normal mice, only a sparse population of CD3^+^, CD8^+^ and CD4^+^ T cells was present in the pancreas (Figure 6j,k; Figures S16 and S17, Supporting Information). In contrast, high‐glucose NOD mice exhibited dense infiltrates of CD3^+^, CD8^+^, and CD4^+^ T cells in both of the edges and islets of the pancreas (Figure 6j,k; Figures S16 and S17, Supporting Information). Post‐treatment, there was a notable decrease in the number of infiltrating CD3^+^ T cells as well as CD8^+^ and CD4^+^ T cells within the pancreas (Figure 6j,k; Figures S16 and S17, Supporting Information). Conversely, Free platelets demonstrated limited efficacy in preventing T cell infiltration (Figure 6j,k; Figures S16 and S17, Supporting Information).

**Figure 7:**
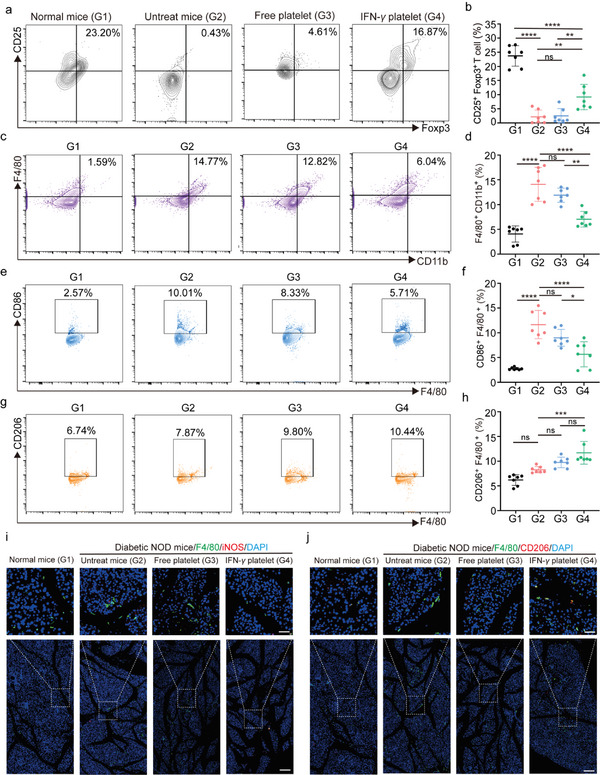
IFN‐*γ* platelets enhance the infiltration of Treg cells and macrophages into the pancreas. a,b) Representative flow‐cytometry data (a) and statistical data (b) to show of CD25^+^ Foxp3^+^ Treg cell infiltration into the pancreas in different groups (CD3^+^CD4^+^ T cells are gated, *n* = 7), G1: Normal mice; G2: Untreat mice; G3: Free platelet; G4: IFN‐*γ* platelet. c,d) Flow cytometry analysis shows the percentage of macrophages in all pancreatic cells (gated for total cells, *n* = 7), G1: Normal mice; G2: Untreat mice; G3: Free platelet; G4: IFN‐*γ* platelet. Error bars: mean ± s.d. e,f) Representative flow‐cytometry data (e) and statistical data (f) to show CD80^+^ F4/80^+^ M1 macrophages (F4/80^+^ cell gated, *n* = 7), G1: Normal mice; G2: Untreat mice; G3: Free platelet; G4: IFN‐*γ* platelet. Error bars: mean ± s.d. g,h) Representative flow‐cytometry data (g) and statistical data (h) to show CD206^+^ F4/80^+^ M2 macrophages (F4/80 cell gated, *n* = 7), G1: Normal mice; G2: Untreat mice; G3: Free platelet; G4: IFN‐*γ* platelet. Error bars: mean ± s.d. i) Immunofluorescence analysis of F4/80 and INOS cells in normal mouse and treated pancreas (scale bar: 200 µm in inserts; 50 µm in enlarged images.). j) Immunofluorescence analysis of F4/80 and CD206 cells in normal mouse and treated pancreas (scale bar: 200 µm in inserts; 50 µm in enlarged images.). Throughout the experiment, NS denotes no significant difference, *p < 0.05, **p < 0.01, ***p < 0.001; a two‐way ANOVA was performed, followed by post‐hoc tests for analysis.

Subsequently, we explored how IFN‐*γ* platelets affect Treg cells. Using flow cytometry, we observed Treg cell infiltration in the pancreatic tissue of NOD mice. The IFN‐*γ* platelets markedly enhanced the accumulation of Treg cells within the pancreatic tissue (**Figure** **7**a,b). In contrast, Treg cell infiltration was intensively reduced in high‐glucose mice, indicating an overactive immune response (Figure 7a,b). Moreover, treatment with IFN‐*γ* platelets significantly boosted the proportion of Treg cells in the pancreatic tissue, contributing positively to the maintenance of immune tolerance (Figure 7a,b). In comparison, Free platelets showed only a minimal impact on the infiltration of Treg cells into the pancreatic tissue (Figure 7a,b). Treg cells can inhibit the activation of effector T cells by secreting suppressive cytokines such as TGF‐*β*, thereby reducing immune attacks on pancreatic *β*‐cell, helping to restore *β*‐cell function, and slowing or reversing the progression of diabetes.^[^ ^63^ ^]^ Treg cells also reduce the destructive effects of the immune system on the islets by suppressing the production of pro‐inflammatory cytokines such as TNF‐*α* and IFN‐*γ*, thereby decreasing the onset and progression of diabetes.^[^ ^63^ ^]^ Tregs are essential for modulating the pancreatic microenvironment and can promote *β*‐cell regeneration and protection through various mechanisms.^[^ ^63^ ^]^ Furthermore, we also assessed the proportion of macrophages in the pancreas and the M1/M2 macrophage ratio. Flow cytometry results showed that IFN‐*γ* platelets notable reduced the proportion of M1 macrophages, and promoted the conversion from M0 to M2 macrophages (Figure 7c–h). In a normal mouse pancreas, both of M1 and M2 macrophages were present at low levels. In contrast, NOD mice with hyperglycemia showed higher levels of M1 macrophage infiltration and lower levels of M2 macrophages at the pancreatic edge and within the islets (Figure 7c–h). After treatment, there was an intensive reduction in M1 macrophages infiltrating the pancreas and a notable increase in infiltrating M2 macrophages (Figure 7c–h). Notably, Free platelets were less effective at preventing M1 macrophage infiltration and promoting M2 macrophage infiltration (Figure 7c–h). Research indicates that in the pancreatic microenvironment, macrophages are crucial for the regeneration of *β*‐cells. This is facilitated through their interactions with both endothelial and pancreatic cells, mediated by signals from TGF‐*β*1 and VEGFR2.^[^ ^64^ ^]^ Previous studies indicate that M2 macrophages promote *β*‐cell proliferation through the release of TGF‐*β*1 and EGF, with the upregulation of SMAD7 in β‐cells further facilitating this process.^[^ ^65^ ^]^ To assess infiltrating macrophages in the pancreas, we collected tissue from NOD mice. Immunofluorescence staining showed that normoglycemic NOD mice exhibited reduced infiltration of M1 and M2 macrophages, whereas hyperglycemic NOD mice had significant M1 macrophage infiltration (Figure 7i,j). After IFN‐*γ* platelets treatment, M1 macrophage infiltration decreased, whereas M2 macrophage infiltration increased (Figure 7i,j). Normal mouse pancreases have low numbers of M1 and M2 macrophages, but hyperglycemic NOD mice showed increased M1 infiltration at the pancreatic edge and in the islets (Figure 7i,j). Post‐treatment, M1 macrophage infiltration significantly decreased, while M2 macrophages increased. In contrast, Free platelets had a limited effect on M1 macrophage infiltration (Figure 7i,j). These data indicated that during treatment, IFN‐*γ* platelets augmented Treg cell magnitude, and promoted M0 macrophages transition to M2 macrophages, which may together promotes *β*‐cell proliferation.

### 2.7 IFN‐*γ* Platelets Promote Pancreatic *β*‐Cell Proliferation

**Figure 8:**
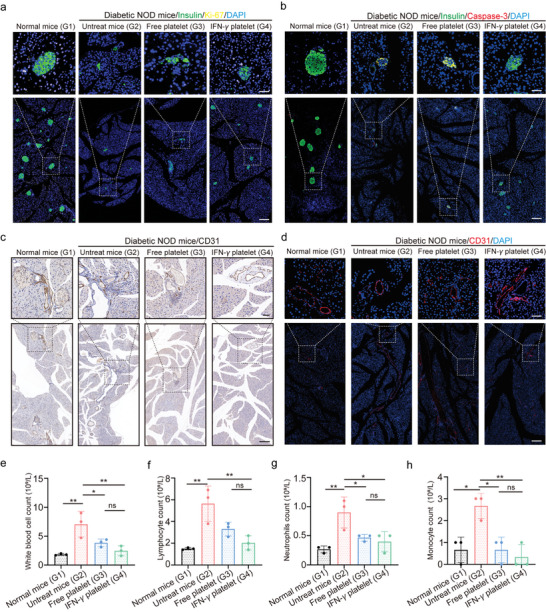
IFN‐*γ* platelets promote the proliferation of pancreatic *β*‐cell and inhibit their apoptosis. a) Immunofluorescence analysis of insulin and Ki‐67 cells in the pancreas of normal mice and after treatment (scale bar: 200 µm in inserts; 50 µm in enlarged images.). b) Immunofluorescence analysis of insulin and Casepase3 cells in the pancreas of normal mice and after treatment (scale bar: 200 µm in inserts; 50 µm in enlarged images.). c) Immunohistochemical analysis of CD31 cells in the pancreas of normal mice and after treatment (scale bar: 200 µm in inserts; 50 µm in enlarged images.). d) Immunofluorescence analysis of CD31 cells in the pancreas of normal mice and after treatment (scale bar: 200 µm in inserts; 50 µm in enlarged images.). e–h) Blood counts show the concentration of white blood cells, lymphocytes, neutrophils, and monocytes at different time points after IFN‐*γ* platelets injection (*n* = 3). Throughout the experiment, NS denotes no significant difference, *p < 0.05, **p < 0.01, ***p < 0.001; a two‐way ANOVA was performed, followed by post‐hoc tests for analysis.

Additional investigations were conducted to determine if the healing benefits of platelets in diabetic mice were due to their influence on *β*‐cell multiplication. By utilizing immunofluorescence staining to identify both single and multiple Ki‐67^+^ insulin^+^ cells within the pancreatic islets, it was observed that administering IFN‐*γ* platelets increased the proliferation of insulin‐producing *β*‐cells in the pancreas (**Figure** **8**a). Additionally, staining of caspase‐3^+^ insulin^+^ *β*‐cells indicates prevention of apoptosis in *β*‐cells following IFN‐γ platelet treatment (Figure 8b). These findings suggest that IFN‐*γ* platelet with multiple immunosuppressive ligands such as PD‐L1 on platelets playing a crucial protective role.

The vascular wall, particularly the endothelium, serves as the primary barrier against the autoimmune destruction of the pancreas mediated by T cells.^[^ ^66^ ^]^ CD31, a specific marker for endothelial cells, is commonly utilized to evaluate angiogenesis induced by injury.^[^ ^67^ ^]^ Immunohistochemistry and immunofluorescence staining analyses revealed a prominent higher number of endothelial cells in IFN‐*γ* platelet treated mice compared to other groups, suggesting that IFN‐*γ* platelet treatment enhanced pancreatic angiogenesis in diabetic mice (Figure 8c,d). Prior research shows that blood vessels in the pancreatic microenvironment support *β*‐cell regeneration via VEGF‐A signaling, which activates pancreatic endothelial cells and recruits macrophages that enhance *β*‐cell proliferation, while also highlighting the potential risk of systemic inflammation caused by blood platelets.^[^ ^68^ ^]^ H&E staining of major organs revealed that platelet infusion via the tail‐vein in mice did not induce any organ damage (Figure S18, Supporting Information). However, routine blood counts are essential for an accurate assessment of the systemic toxicity of IFN‐*γ* platelet therapy. After 60 days of treatment, immune cell counts in the blood were measured. Following complete blood count testing, an intensive reduction in immune cell numbers was observed in the blood of NOD mice treated with IFN‐*γ* platelets. However, in hyperglycemic mice, the levels of white blood cells, lymphocytes, neutrophils, and monocytes were notable elevated, indicating an overactive immune response (Figure S19, Supporting Information). Conversely, treatment with IFN‐*γ* platelets led to an intensive reduction in these immune cell levels, contributing to the maintenance of immune tolerance. In contrast, Free platelets had a limited effect on the levels of these immune cells (Figure 8e–h). Furthermore, a hemolysis test was performed to evaluate platelet safety. The results shown that both Free platelets and IFN‐*γ* platelets exhibited a hemolysis rate of less than 5% (Figure S19, Supporting Information). These data suggest that IFN‐*γ* platelet treatment remarkable enhances pancreatic *β*‐cell proliferation, stimulates angiogenesis, and promotes pancreatic repair in T1D mouse model.

## 3 Discussion

The attack by autoimmune T cells directly leads to *β*‐cell impairment.^[^ ^7^ ^]^ Contemporary investigations into type 1 diabetes aim to develop novel strategies for the targeted elimination of autoimmune T cells, moving beyond conventional insulin therapy.^[^ ^69^ ^]^ Notably, herapy involving immune checkpoint blockade not only shows considerable promise in treating cancer but also plays a crucial role in maintaining immune tolerance in healthy tissues and organs.^[^ ^70^ ^]^ For instance, mice that lack the CTLA‐4 gene exhibit severe autoimmune reactions that affect multiple organs, demonstrating the gene's role in immune regulation. Similarly, disrupting the PD‐1/PD‐L1 signaling pathway can induce various autoimmune disorders. Among these is T1D, showcasing how crucial this pathway is for maintaining immune homeostasis and preventing autoimmune diseases.^[^ ^27^ ^]^ Furthermore, studies have used HAL‐loaded MSC exosomes with high levels of the immune checkpoint molecule PD‐L1 to show strong immunosuppressive and anti‐inflammatory effects in T1D models.^[^ ^36^ ^]^ Numerous studies have demonstrated that drugs overexpressing PD‐L1 exhibit significant immunosuppressive and anti‐inflammatory effects in type 1 diabetes models, thereby enhancing the overall condition of the disease.^[^ ^34^, ^36^, ^38^, ^39^ ^]^ To further elucidate the therapeutic effects of inhibitory immune checkpoints, we propose a novel approach to multi‐immune checkpoint blockade therapy by combining PD‐L1, PD‐L2, BTLA, and Gal‐9 four inhibitory immune ligands for the treatment of T1D. These inhibitory immune checkpoint ligands suppress autoimmune response‐inducing immune cells and enhance Treg cell generation and function, contributing to T1D management. Additionally, Gal‐9 interacts with TIM‐3, which promote Treg cell differentiation, proliferation and inhibit Th1 cell effector functions, thereby aiding in the establishment and maintenance of immune tolerance.^[^ ^71^ ^]^ Furthermore, Treg cells modulate immune responses by secreting immunomodulatory factors and inhibiting other immune cell activities in the pancreatic microenvironment, thereby promoting pancreatic *β*‐cell regeneration and protection.^[^ ^72^ ^]^ Tregs are essential for maintaining immune homeostasis as they directly suppress T cell activity. Various mechanisms are employed to induce immunosuppression, including the secretion of anti‐inflammatory cytokines, namely IL‐10 and TGF‐*β*. These cytokines inhibit both the proliferation and the activity of effector T cells. Furthermore, Tregs can influence the immune response through direct cell–cell interactions with other immune cells, employing surface molecules like CTLA‐4 and PD‐1 to inhibit T cell activation. Our engineered platelets promote Treg proliferation by releasing immunoregulatory factors like IL‐10 and TGF‐*β* upon activation, enhancing Treg functionality. Additionally, direct contact between platelets and Tregs may stimulate Treg activation and proliferation via surface molecules like CD40L and CD62P.^[^ ^73^ ^]^

The strong anti‐inflammatory and inflammation‐targeting properties of M2 macrophages have attracted researchers' interest in combining them with other immunotherapies. M2 macrophages suppress T cell activity through several mechanisms. At the outset, these cells release anti‐inflammatory cytokines like IL‐10 and TGF‐*β*. These substances are effective in inhibiting the proliferation of T cells and reducing cytokine production.^[^ ^74^, ^75^, ^76^ ^]^ Additionally, M2 macrophages express immune checkpoint molecules like PD‐L1 on their surface, which interact with corresponding receptors on T cells, thereby inhibiting their activity.^[^ ^77^ ^]^ Furthermore, M2 macrophages can modify the local microenvironment by secreting metabolic products such as lactate, thereby further inhibiting T cell function.^[^ ^77^ ^]^ These mechanisms collectively allow M2 macrophages to play a critical role in regulating immune responses. Moreover, under inflammatory conditions, exosomes derived from mesenchymal stem cells (MSCs) have been shown to improve cell migration and support the differentiation of M2 macrophages. This contributes to enhanced therapeutic results in rabbit models of cartilage defects.^[^ ^78^ ^]^ Recent research suggests that M2 exosomes (Exos), engineered from M2 macrophages, are highly effective for both imaging and treating atherosclerosis. Their natural anti‐inflammatory qualities and targeted action on inflammation underscore their significant potential.^[^ ^79^ ^]^ These EVs induce M1 macrophages to polarize into an M2 phenotype, adjust the immune microenvironment, and show highly effective therapeutic effects in T1D mouse models.^[^ ^36^ ^]^ Our engineered platelets can promote the polarization of M0 macrophages to M2, while M2 macrophages suppress T cell activity through several mechanisms. Furthermore, M2 macrophages promote *β*‐cells proliferation by secreting TGF‐*β*1 and EGF.^[^ ^65^ ^]^ Additionally, increasing SMAD7 levels in β‐cells can also boost their proliferation. Thus, growth factors from M2 macrophages and SMAD7 are key to promoting *β*‐cells proliferation.^[^ ^65^ ^]^ M2 macrophages are crucial in mitigating inflammation, aiding tissue reconstruction, and adjusting immune reactions through the release of factors like IL‐10 and TGF‐*β*. These cells also express surface molecules such as PD‐L1 and PD‐L2.^[^ ^74^, ^75^, ^76^, ^77^ ^]^ Studies indicate that the activation of the PDGFR pathway significantly impacts the growth of *β*‐cells within pancreatic islets in both mice and humans. This growth promotes the expansion of *β*‐cells and plays a role in potentially reversing diabetes.^[^ ^59^ ^]^ Therefore, our engineered platelets may also enhance the proliferation of β‐cells via SMAD7 levels and the PDGFR signaling pathway.

Endothelial cells have strong regenerative capabilities, leading researchers to explore combining them with other immunotherapies as a new research and treatment approach. The vascular wall, especially the endothelial layer, acts as a crucial barrier against autoimmune T cell damage to the pancreas.^[^ ^66^ ^]^ Blood vessels in the pancreatic microenvironment produce VEGF‐A signals that work with endothelial cells (ECs) in the pancreatic vasculature to promote *β*‐cell regeneration.^[^ ^68^ ^]^ VEGFR2 regulates the VEGF‐A signal to activate pancreatic vascular endothelial cells, which leads to *β*‐cell loss but helps maintain the stability and quality of both pancreatic vasculature and *β*‐cells.^[^ ^68^ ^]^ VEGF‐A induction recruits CD45^+^CD11b^+^Gr1^+^ macrophages, which remain in pancreatic remnants after VEGF‐A stimulation stops, producing effector molecules that promote *β*‐cell proliferation and regeneration regardless of pancreatic location and systemic factors.^[^ ^68^ ^]^ Recognizing the platelets components is pivotal for *β*‐cell development, function, and homeostasis in the pancreatic microenvironment, we validated the impact of IFN‐*γ* platelets on angiogenesis in the pancreas. Hence, our engineered platelet treatment results indicate that the engineered platelets may enhance pancreatic angiogenesis as well as *β*‐cell proliferation and regeneration in diabetic mice. Looking ahead, several key scientific questions remain, including how to optimize platelet engineering for enhanced therapeutic efficacy and how to better understand the long‐term effects of IFN‐*γ* platelet treatment on immune responses and organ function. Potential technological improvements could include the development of more efficient methods for platelet modification and targeted delivery. Moreover, future clinical trial designs should focus on assessing the safety, efficacy, and optimal dosing of IFN‐*γ* platelet treatments in human subjects, aiming to establish new therapeutic strategies for autoimmune diseases like Type 1 diabetes.

## 4 Conclusion

In summary, we engineered platelets to enrichment of the immune‐suppressive ligands PD‐L1, PD‐L2, BTLA, and Gal‐9. Virtually, IFN‐*γ* platelets could efficaciously induce T cell exhaustion, even elicited T cell apoptosis in vitro. Intriguingly, IFN‐*γ* platelets treatments intensively reduced T cell infiltration in the pancreas and prominently augmented the generation of Tregs, which conducive to *β*‐cell integrity and hindered the progress of T1D. Moreover, IFN‐*γ* platelets converted the macrophage polarization toward an anti‐inflammatory M2 phenotype, which might contribute to stimulate pancreatic angiogenesis, and enhanced *β*‐cell proliferation and regeneration.

## Conflict of Interest

The authors declare no conflict of interest.

## Author Contributions

Y.M. and F.M. contributed equally to this work. X.Z. and X.L. designed the study. Y.M., F.M., Z.L., Y.C., T.L., Z.Y., R.D., X.Z., Q.C., C.Z., Y.T., C.L., and W.F. performed the experiments. Y.M. and F.M. interpreted the data and wrote the manuscript. The manuscript was written through the contributions of all authors. All authors have given approval to the final version of the manuscript.

## Supporting information



Supporting Information

## Data Availability

Research data are not shared.
