# Supplementary material for: Bioengineering Platelets Presenting PD‐L1, Galectin‐9 and BTLA to Ameliorate Type 1 Diabetes
Source: Adv Sci (Weinh). 2025 Feb 28;12(16):2501139. doi: 10.1002/advs.202501139 (PMC12021092; doi:10.1002/advs.202501139)
Supplement: Supplementary file 1 — Supporting Information [file ADVS-12-2501139-s001.docx]

**Supporting Information**

**Bioengineering Platelets Presenting PD-L1, Galectin-9 and BTLA to Ameliorate Type 1 Diabetes**

Yumeng Ma^1,2,#^, Fanqiang Meng^1,2,#^, Zhongda Lin^1,2^, Yanjun Chen^1,2^, Tianyu Lan^1,2^, Zhaoxin Yang^1,2^, Rui Diao^1,2^, Xiaozhou Zhang^1,2^, Qi Chen^3,4^, Chi Zhang^1,2^, Yishi Tian^1,2^, Chanjuan Li^1,2^, Wenli Fang^1,2^, Xin Liang^3,4,*^, Xudong Zhang^1,2,*^

1. Shenzhen Key Laboratory for Systems Medicine in Inflammatory Diseases, School of Medicine, Shenzhen Campus of Sun Yat-Sen University, Sun Yat-Sen University, Shenzhen, 518107, Guangdong, China.

2. Department of Pharmacology, Molecular Cancer Research Center, School of Medicine, Shenzhen Campus of Sun Yat-sen University, Sun Yat-sen University, Shenzhen 518107, Guangdong, China.

3. Guangdong Provincial Key Laboratory of Medical Molecular Diagnostics, Key Laboratory of Stem Cell and Regenerative Tissue Engineering, School of Basic Medical Sciences, Guangdong Medical University, Dongguan 523808, China.

4.The Affiliated Dongguan Songshan Lake Central Hospital, Guangdong Medical University，Dongguan, Guangdong, 523806, P.R.China.

*.Correspondence: [zhangxd56@mail.sysu.edu.cn](mailto:zhangxd56@mail.sysu.edu.cn；) (Z.X.),

[liangxingibh@gdmu.edu.cn](mailto:liangxingibh@gdmu.edu.cn) (L.X.)

^#^.These authors contributed equally.

**Methods**

1. *Cell culture*: L8057 cells were cultured using RPMI 1640 medium supplemented with 20% fetal bovine serum (FBS). RAW264.7 cells were cultured in DMEM medium that was supplemented with 20% fetal bovine serum (FBS). The mouse CD4^+^ T cell CL.ly1^+^2^−^/9 cells lines were purchased from the National Infrastructure of Cell Line Resource and cultured in RPMI 1640 with 10% FBS with 100 IU mL^−1^ murine interleukin 2. All cells were maintained at 37 °C in a 5% CO_2_ atmosphere during incubation.

1. *Production of platelets*: IFN-*γ*-L8057 cells, along with L8057 cells, were cultured in RPMI 1640 medium and stimulated with 500 nM PMA for 3 days to induce maturation. The matured L8057 cells undergo differentiation and release platelets in the subsequent days. The collected culture medium was centrifuged at 1200 rpm for 20 minutes, after which the precipitate was discarded. The supernatant was then centrifuged at 12,000 rpm for an additional 20 minutes. The platelet pellet was finally resuspended carefully in PBS containing 1 µM PGE1 or in Tyrode’s buffer (1 mM MgCl_2_, 12 mM NaHCO_3_, 2.9 mM KCl, 134 mM NaCl, 0.34 mM Na_2_HPO_4_, 10 mM HEPES, pH 7.4).
2. *Immunofluorescent assay*: L8057 cells were fixed in 4% paraformaldehyde and subsequently washed with PBS. To block and permeabilize the cells, 0.2% Triton X-100 in 3% BSA buffer was used. After that, L8057 cells were incubated with primary antibodies, including CD41, CD42, GPVI, and P-Selectin, for 2 hours at room temperature. After the incubation, the cells were washed with PBS three times. Subsequently, the rhodamine-conjugated secondary antibody, diluted in 1.5% BSA, was used to stain the cells, and the samples were placed in the dark for 1 hour. The nucleus was then stained with DAPI for 20 minutes. Finally, the cells were washed with PBS three times.
3. *PMPs detection*: To detect the release of PMPs from the platelets *in vitro*, the platelets were treated with or without thrombin (1 U/mL) for 60 minutes. The platelets were then analyzed using Dynamic Light Scattering (DLS) and observed with confocal microscopy (Zeiss). To detect the release of PMPs from the platelets *in vivo*, IFN-*γ* platelets were injected via the tail vein into NOD mice. Plasma samples from the NOD mice were collected at various time points as indicated. The platelets and PMPs were then isolated and observed under confocal microscopy.
4. *Platelet TGF-β1 detection*:To detect the secretion of TGF-*β*1, free platelets derived from L8057 cells (1 mg), IFN-*γ* platelets (1 mg), and the cell culture medium were collected. Thrombin (0.5 U/mL) was used to stimulate the platelets for 30 minutes. To disrupt the platelets, 0.5% Triton X-100 was added. The TGF-*β*1 levels in the cell culture medium, active platelets supernatant, and disrupted platelets supernatant were measured using a TGF-*β*1 ELISA kit (MULTI SCIENCES) according to the manufacturer’s instructions.

1. *In vivo biodistribution analysis*: The isolated platelets were labeled with NHS-Cy5.5 in PBS buffer. Diabetic NOD mice and normal mice were injected with Cy5.5-labeled IFN-*γ* platelets and free platelets (200 µL, 250 µg) via the tail vein as indicated. Then, the NOD mice were euthanized at different time points as indicated, and the major organs, including the pancreas, lungs, heart, kidneys, spleen, and liver, were collected. The fluorescence intensity of these major organs was measured using the Xenogen IVIS Spectrum imaging system.
2. *Diabetic NOD mice treatment*: Female NOD/ShiLtJ mice were purchased from Jiangsu Jicui Yaokang Biotechnology Co., Ltd. NOD mice were classified as diabetic when their blood glucose levels exceeded 250 mg/dL^−1^ for two consecutive days. Blood glucose levels in NOD mice were monitored starting at 10 weeks of age. Once hyperglycemia (blood glucose levels > 250 mg/dL^−1^) was observed for two consecutive days, the hyperglycemic mice were either left untreated (control group) or injected with Free platelets (250 mg) or IFN-*γ* platelets (250 mg) via the tail vein every two days. Glucose levels were monitored every two days until the endpoint (60 days), after which the mice were sacrificed for further analysis.
3. *Pancreas T cell analysis*: To evaluate the status of infiltrating T cells in the pancreas, pancreatic tissue was collected from NOD mice subjected to different treatments as indicated. The pancreas was dissociated to generate single-cell suspensions, and the samples were passed through a 70-micron filter. Subsequently, the cells were stained with APC-conjugated anti-CD3, PE-conjugated anti-CD4, Violet660-conjugated anti-CD4，PE-conjugated anti-CD8, Violet660-conjugated anti-CD25，APC-conjugated anti-FoxP3, PE-conjugated anti-Granzyme B, PB450-conjugated anti-IFN-*γ*, FITC-conjugated anti-CD11B, BV510-conjugated anti-F4/80, APC-A750-conjugated anti-cd80, PE-conjugated anti-CD86, and PECY7-conjugated anti-CD206 as indicated. The percentages of CD3^+^CD8^+^ T cells, CD3^+^CD4^+^ T cells, Granzyme B^+^CD8^+^ T cells, and IFN-*γ* ^+^ CD8^+^ T cells, IFN-*γ* ^+^ CD4^+^ T cells, FoxP3^+^CD25^+^CD4^+^ Treg cells, F4/80^+^CD11B^+^ macrophage cell were determined by flow cytometry.
4. *The Binding Assay of Cell with platelets*: Cl.Ly1^+^2^−^/9 cells were seeded in confocal wells. CSFE labeled platelets were added and incubated with Cl.Ly1^+^2^−^/9 cells for 4 h.DAPI was then added to label the cell nuclei for 12 minutes. Following this, the cells were observed using confocal microscopy (Zeiss).
5. *CO-IP Assa*y: CL.ly1+2−/9 cells (1×10^7^ ) were incubated with IFN-*γ* platelets (300 µg) for 12 h. The cells were washed three times with PBS and then lysed on ice using cold RIPA buffer for 30 minutes. The lysate was centrifuged at 15,000 g for 10 minutes at 4°C. The supernatant was incubated overnight at 4°C with primary antibodies against PD-1, TIM-3, and HVEM. On the following day, 10 µL of protein A/G-agarose beads were added to the lysate and incubated for 2 hours at 4°C. The lysate was then centrifuged at 15,000 g for 5 seconds at 4°C, and the pellet was washed gently with cold RIPA buffer three times. Finally, the bound proteins were collected and subjected to Western blot analysis using antibodies against PD-L1, PD-L2, BTLA, and GAL-9.

11.*In Vivo Biodistribution Analysis*: Cy5.5-conjugated free platelets and IFN-*γ* platelets were prepared and washed with PBS for 3 times to remove the unconjugated Cy5.5. With 200 µL Cy5.5-labeled IFN-*γ* platelets were delivered into the MRL/lpr mice (n = 3 per group) via tail-vein injection. Free platelets were used as a control. After 12, 24, and 48 hours, the mice were sacrificed, and vital organs were homogenized in PBS buffer. Finally, each organ was measured for radiant efficiency calculation. Radiant efficiency was acquired using Living Image software.

12.*Tissue Immunofluorescent Assay*: The pancreas samples were initially fixed in 4% paraformaldehyde overnight and then frozen in optimal cutting temperature (OCT) compound. The frozen tissues were sectioned into 6 μm thick slices and adhered to glass slides. The pancreas slices were washed in PBS for 15 minutes to remove the OCT compound and then boiled in antigen retrieval solution for 20 minutes. After natural cooling-off, the slides were incubated with 3% BSA for 1h then incu bated with CD4 antibody (1:100 dilution, abcam, ab183685), CD8 antibody (1:100 dilution, abcam, ab22378) or CD11c antibody (1:100 dilution, abcam, ab33483) at 4 ℃ overnight. The slices were washed in PBS for three times and incubated with Alexa Fluor® 488-conjugated Goat Anti-Rabbit IgG (1:200 dilution, abcam, ab150077), Alexa Fluor® 594-conjugated Goat Anti-Rat IgG (1:200 dilution, abcam, ab150160) or Alexa Fluor® 488-conjugated Goat Anti-Armenian Hamster IgG (1:200 dilution, abcam, ab173003) for 1 h at room temperature. Finally, the slices were washed in PBS three times and stained with DAPI for 10 minutes to label the nuclei. All samples were analyzed using confocal microscopy (Zeiss).

13.*Transmission Electron Microscope (TEM)*: Extract a 10 μl sample and pipette it onto a copper grid, allowing it to settle for 1 minute. Remove any excess liquid using filter paper. Next, add 10 μl of uranyl acetate (or phosphotungstic acid staining solution) onto the copper grid and let it settle for another minute. Remove the excess liquid with filter paper and air-dry the grid at room temperature for several minutes. Perform electron microscopy imaging at 80-120 kV, and observe and collect images for analysis using transmission electron microscopy.

14.*Platelet size detection*: Add 100 ng/mL of platelets to a cuvette and measure the platelet size. To detect platelet microparticles (PMP) produced after activation, activate platelets with thrombin (0.5 IU^-1^) for 60 seconds, then measure the size of platelet microparticles.

15. *SDS-PAGE assay*: The recombinant protein from L8057 cells and platelets was boiled with 1× loading buffer and then separated using a 10% sodium dodecyl sulfate polyacrylamide gel electrophoresis (SDS-PAGE) gel. The protein was then transferred onto a polyvinylidene fluoride (PVDF) membrane, which was blocked with 5% skimmed milk in tris buffer solution with Tween 20 (TBST, 0.1%) for 2 hours at room temperature. The membrane was hybridized with PD-L1, PD-L2, BTLA, TGF-*β*1, CD73, Nectin-2, SIRP-*α*, VTCN1 or IDO antibodies (1:1000) overnight at 4◦C. The membrane was then washed three times for approximately 15 minutes each with TBST buffer and incubated with a secondary antibody for 1 hour at room temperature. After three washes of 5 minutes each with TBST, imaging was performed using enhanced chemiluminescence detection (ECL, FDbio-Pico).

1. *In vitro promotion of macrophages:* RAW264.7 cells were plated on 6-well plates and cultured until reaching a density of 60%. Free platelets and IFN-*γ* platelets were then added, and the cells were incubated for 24 hours. Subsequently, the cells were stained with FITC-conjugated anti-CD11B, BV510-conjugated anti-F4/80, and PECY7-conjugated anti-CD206 as indicated.The percentages of CD206^+^ F4/80^+^ macrophage cell were determined by flow cytometry.
2. *HE Staining*: Dewax the paraffin sections to water, then wash with tap water. Immerse the sections in Hematoxylin Staining Solution A for 3-5 minutes, wash with tap water, differentiate with Hematoxylin Differentiation Solution B for 3-5 seconds, wash with tap water, and then blue with Hematoxylin Bluing Solution C for 3-5 seconds. Rinse with running water. Dehydrate the sections sequentially in 85% and 95% alcohol for 5 minutes each, and then stain in Eosin Staining Solution D for 3-5 minutes.Next, place the sections in absolute ethanol I for 5 minutes, absolute ethanol II for 5 minutes, absolute ethanol III for 5 minutes, Dimethyl I for 5 minutes, and Dimethyl II for 5 minutes for transparency. Finally, mount with neutral balsam. Examine under a microscope, and capture and analyze the images.

18*. Statistical analysis*: All data results are based on the mean ± standard deviation (mean

± SD). The statistical software SPSS 16.0 was used to compare the differences in each group. Student’s t-test was used for analyzing the differences between two groups. One-way analysis of variance (ANOVA) was used for analyzing the differences between three or more groups, and Tukey was used for test correction. The survival curves were compared with Log-Rank (Mantel-Cox) analysis. *P < 0.05, **P < 0.01, ***P < 0.001, ****P < 0.0001. The used statistical method was indicated in the figure legend. The all n in the figure legends represented the number of biological replicates.


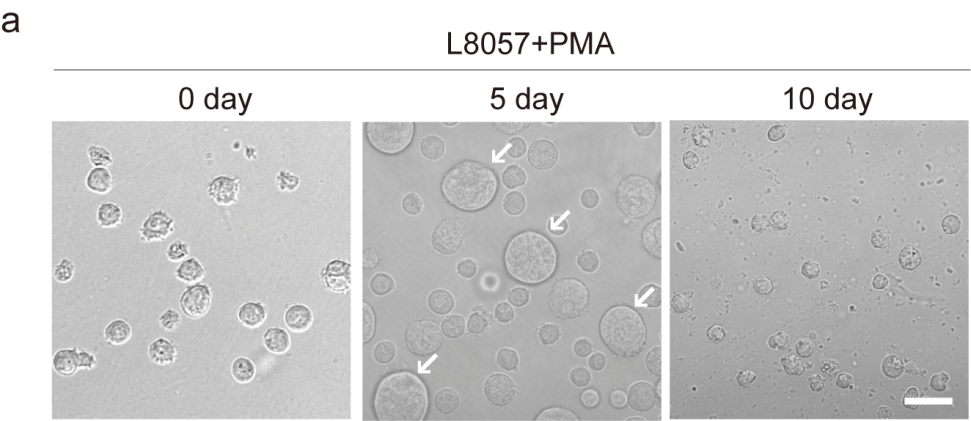


**Figure S1**. Platelets released from L8057 cells. L8057 cells were mature and differentiated after treatment with 500 nM PMA for 0, 5 and 10 days, respectively. Scale bar: 10 µm.


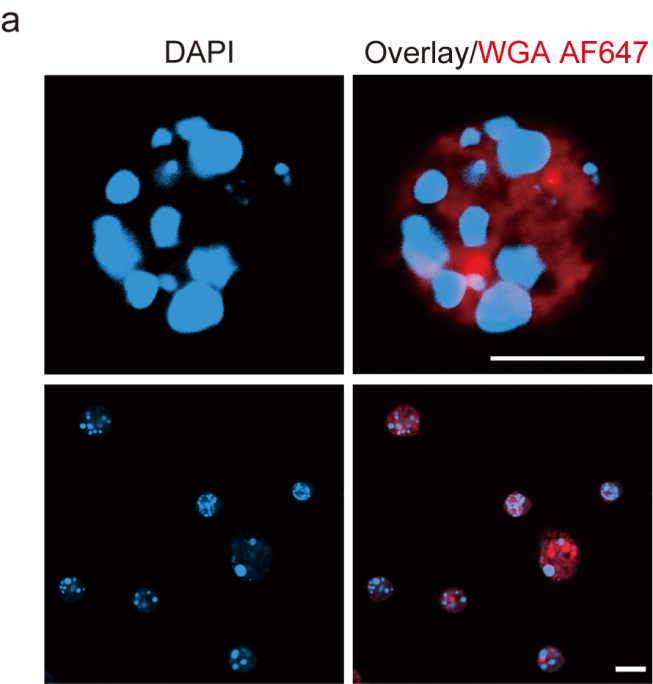


**Figure S2**. Confocal image of mature L8057 cells containing polyploid nuclei after treatment with 500 nM PMA. Scale bars: 10 µm.


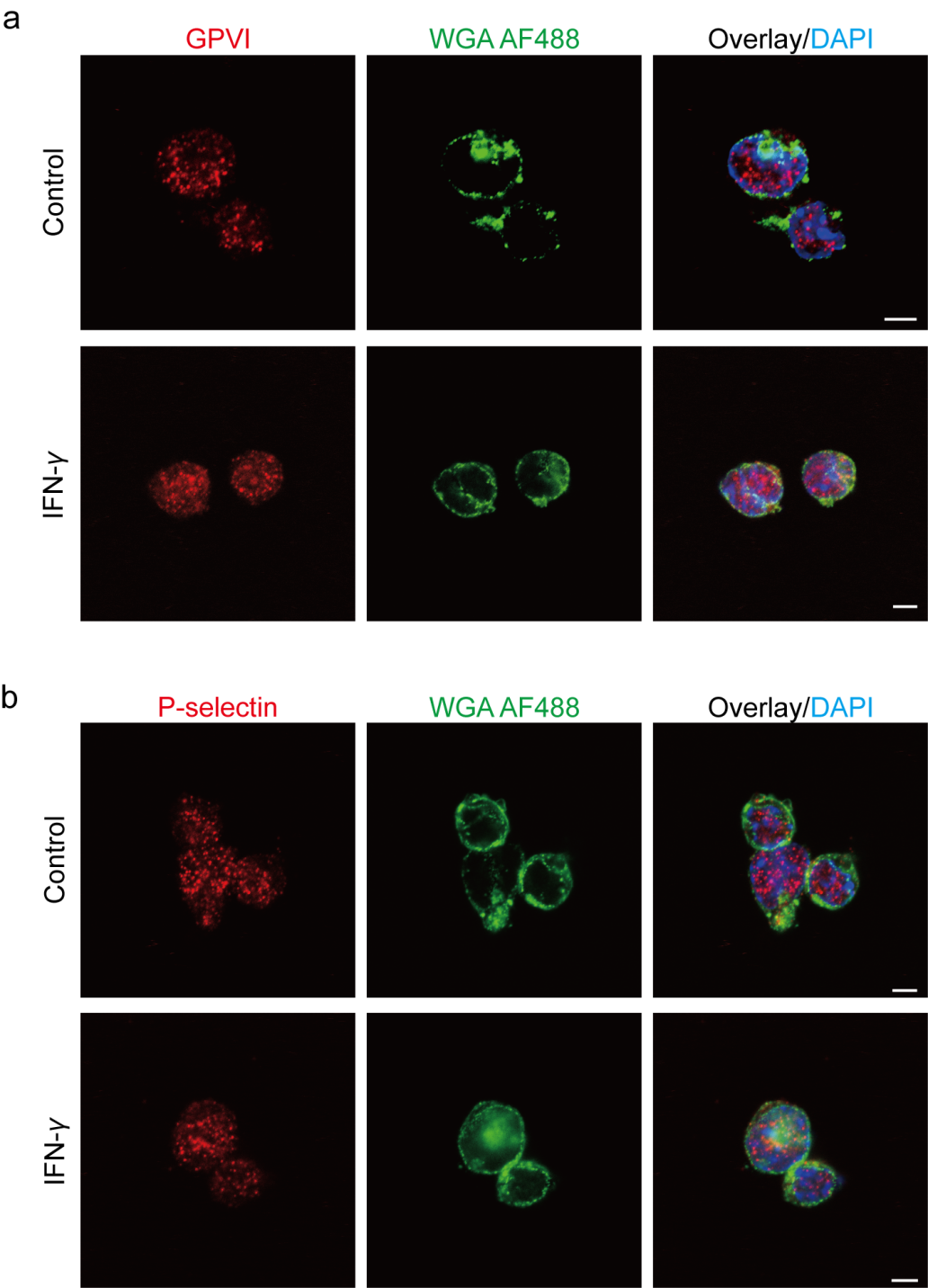


**Figure S3**. Maturation and biomarker of MKs. Representative confocal images of L8057 cells stained for the detection of P-Selectin and GPVI expression. Scale bars: 5 µm.


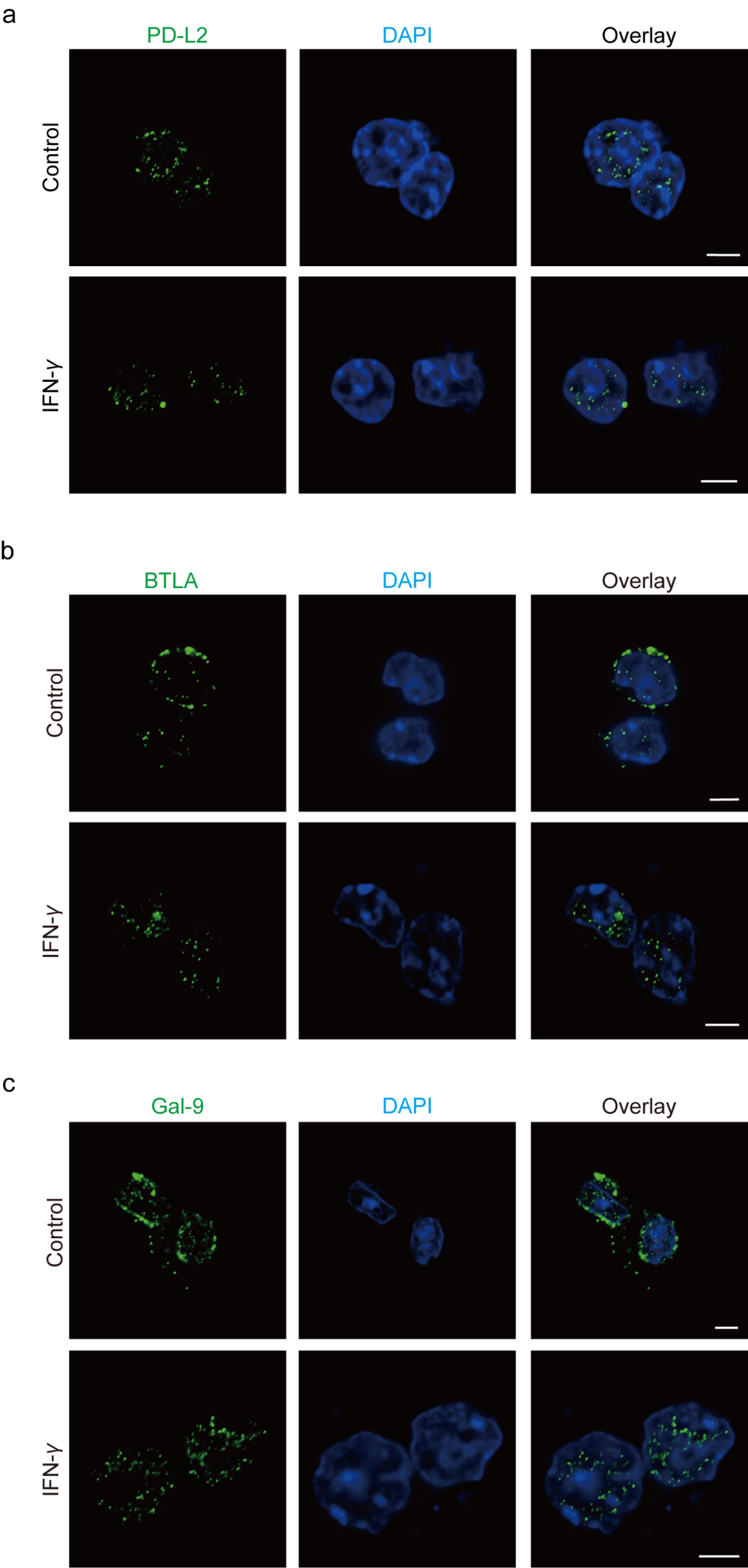


**Figure S4.** Immunofluorescence staining was used to detect the expression of PD-L2, BTLA, and GLA-9 in L8057 cells before and after IFN-*γ* stimulation. Scale bar: 5 µm.


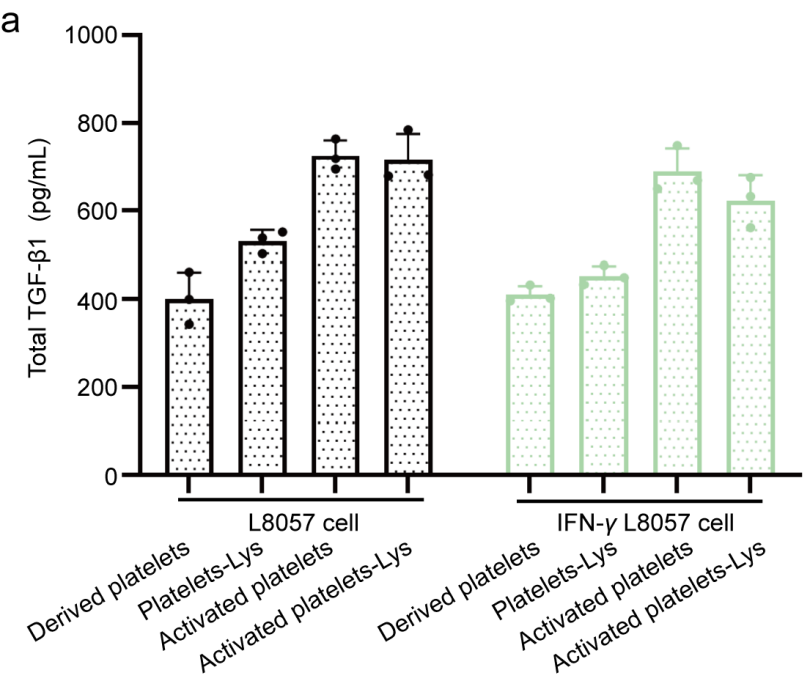


**Figure S5**. Total TGF-*β*1 levels in L8057 cells and IFN-*γ* L8057 cells derived platelets (1mg) lysates (Lys) and platelets releases (Rel) were measured by ELISA (*n*=3).

**
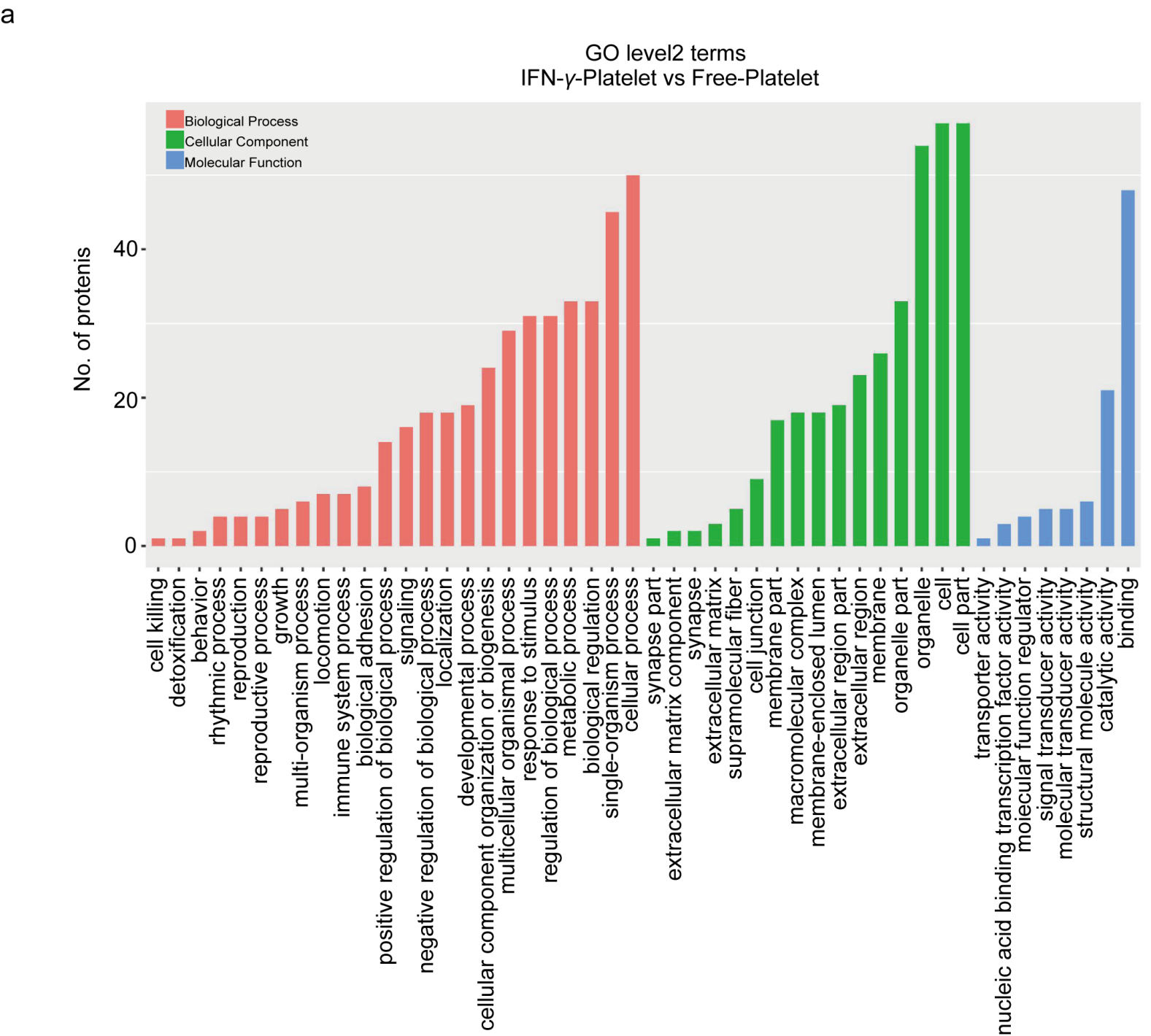
**

**Figure S6.** The Gene Ontology (GO) annotation of differentially expressed proteins between Free-Platelet and IFN-*γ*-Platelet.

**
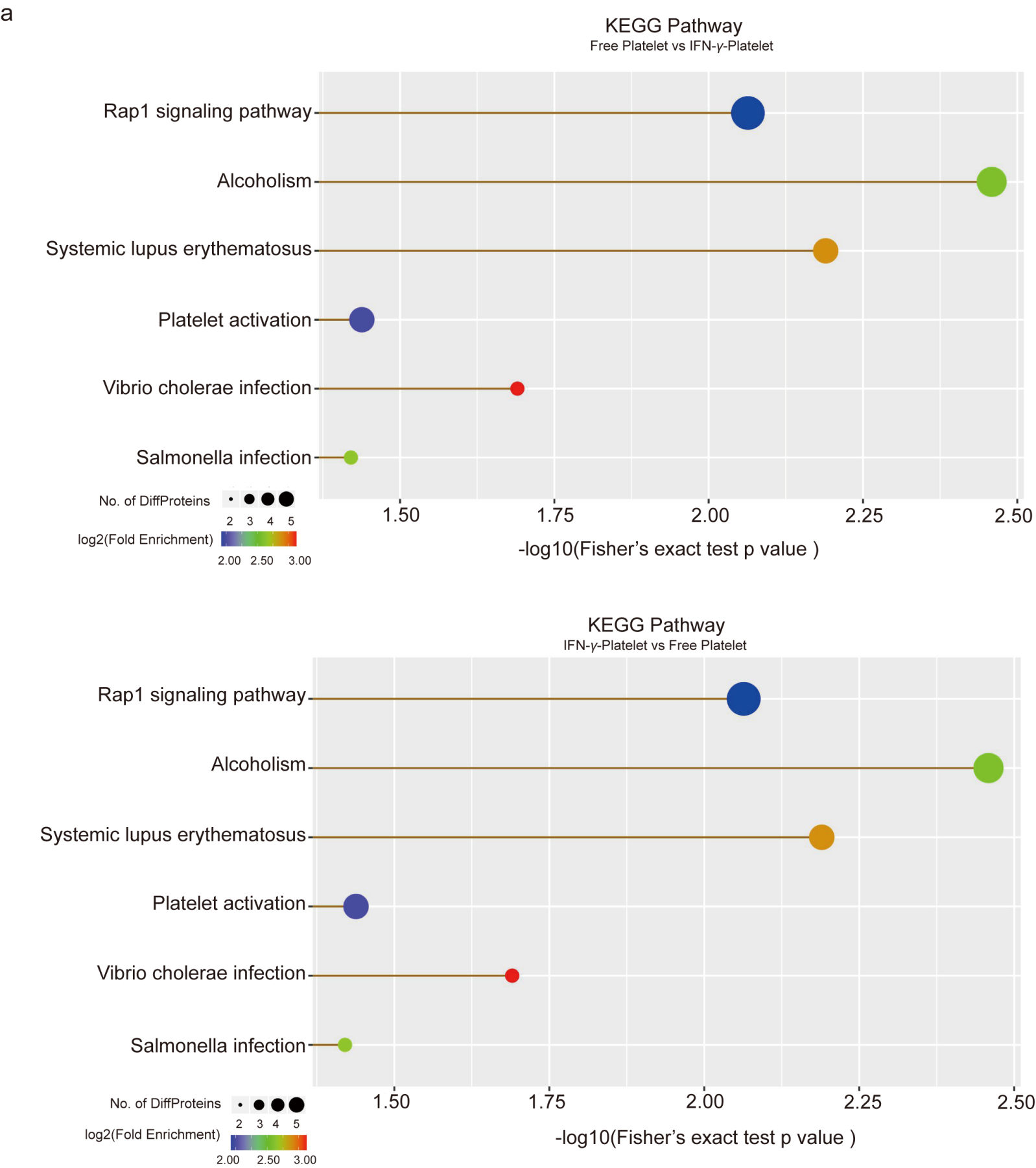
**

**Figure S7.** KEGG pathways based on pathway enrichment analysis of differentially expressed proteins.

**Figure S8.** Differential protein function clustering of cellular components based on differentially expressed proteins.

**Figure S9.** Clustering of differential protein functions within cellular components based on the expression patterns of differentially expressed proteins

**Figure S10.** Biological processes were elucidated through pathway enrichment analysis of differentially expressed proteins.


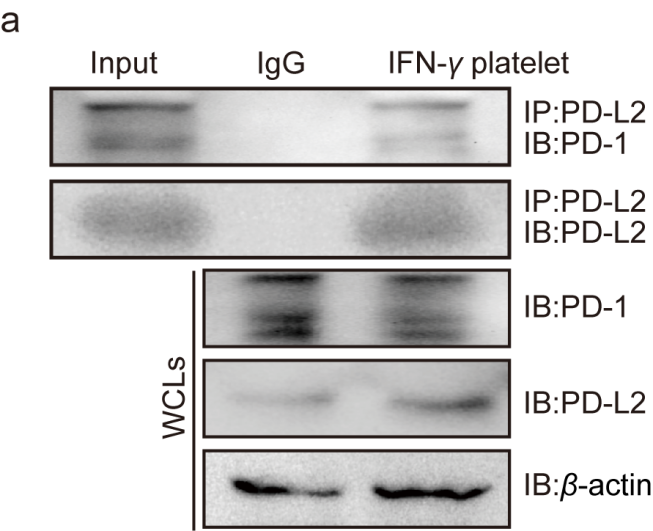


**Figure S11.** The coimmunoprecipitation assay was used to investigate the interaction between PD-L2 on IFN-*γ* platelets and PD-1 on CI.Ly1^+^2^−^/9 cells.


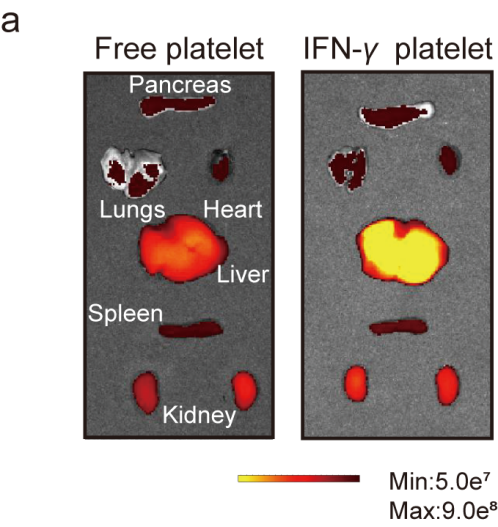


**Figure S12.** After intravenous administration of Free platelets and IFN-*γ* platelet, fluorescence spectroscopic imaging was performed to visualize their biodistribution in major organs and the pancreas.


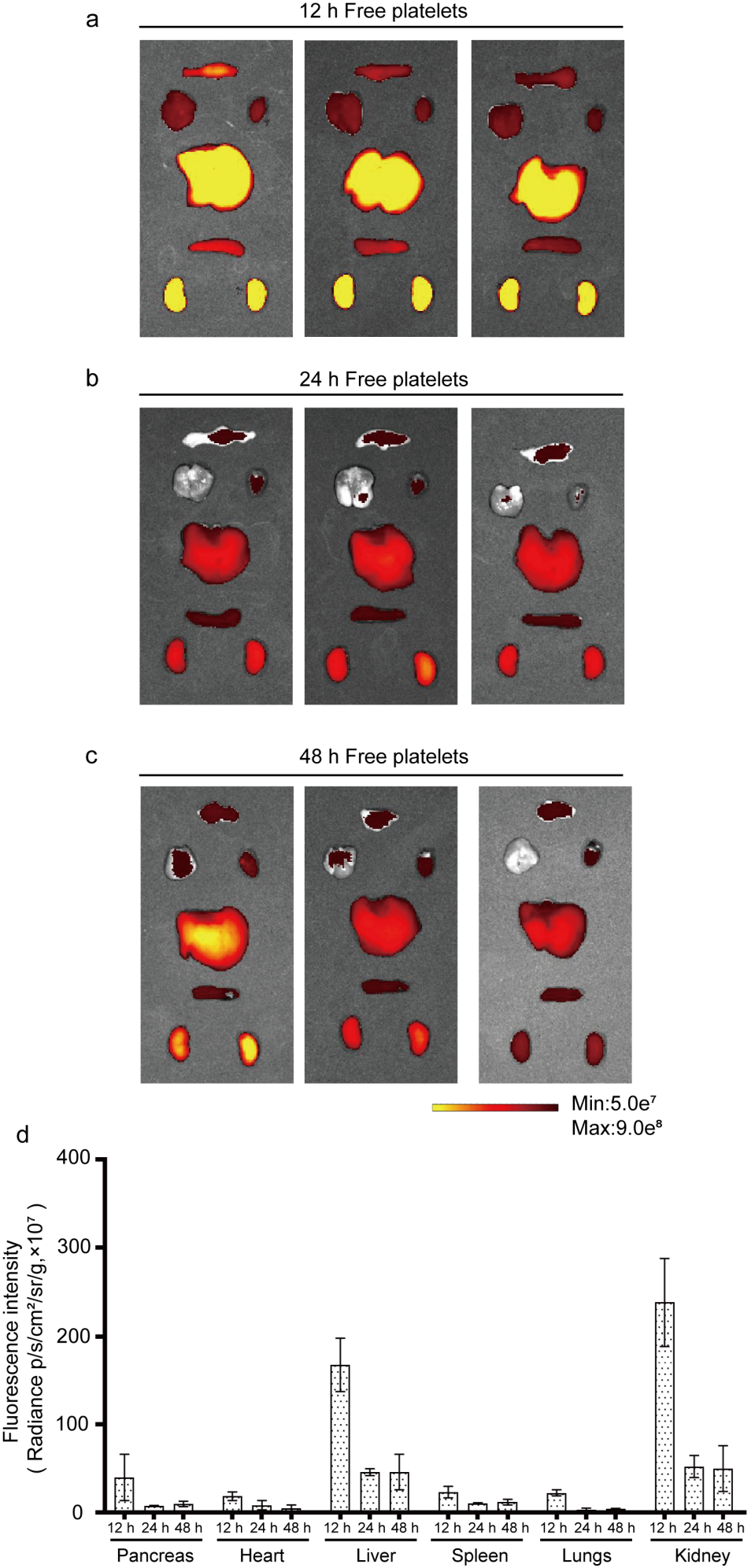


**Figure S13**. Free platelets vivo fluorescence biodistribution of vital organs (heart, liver, spleen, lung, kidney, and pancreas) at 12, 24, and 48 h post-injection.


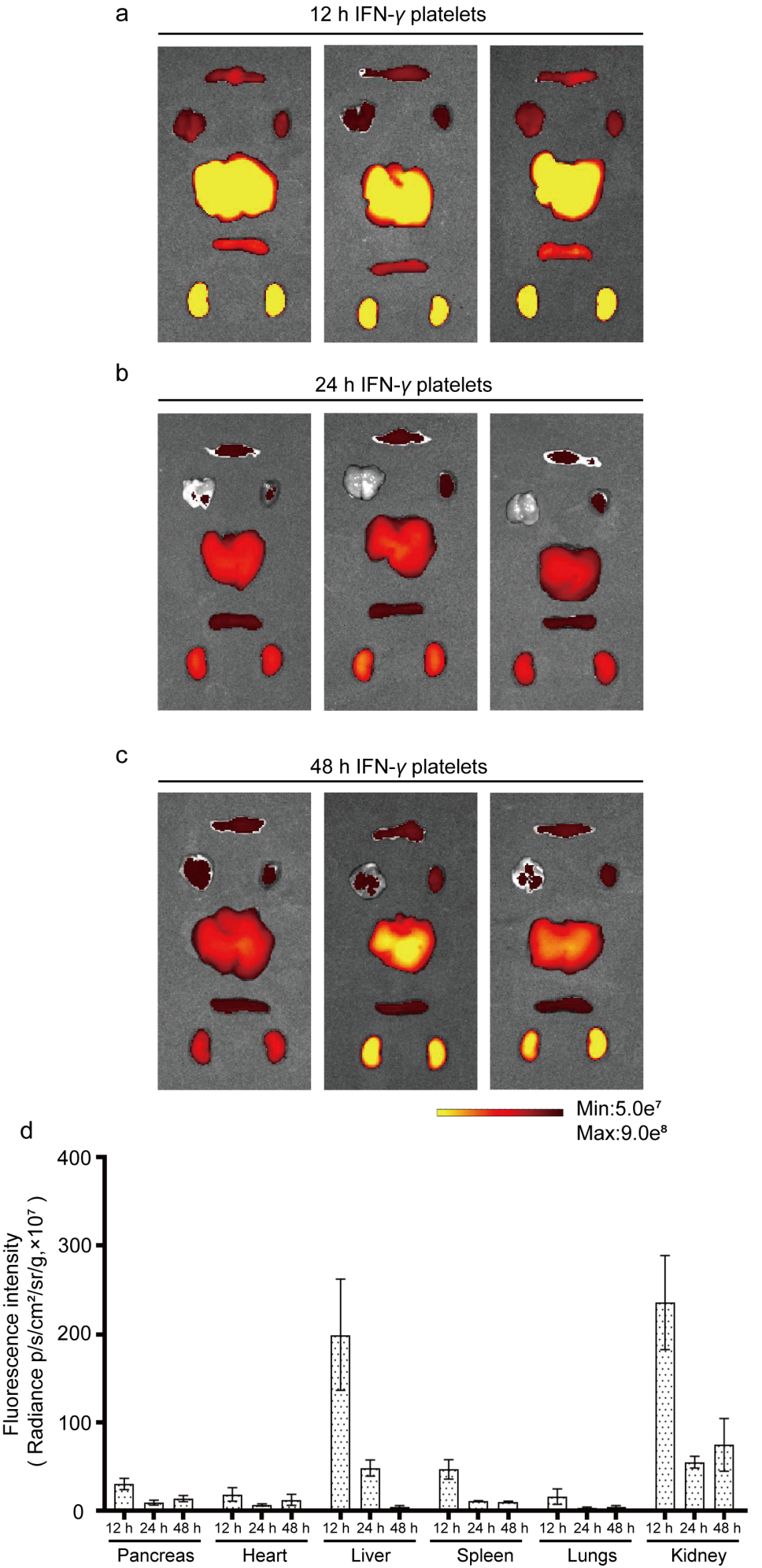


**Figure S14**. IFN-*γ* platelets vivo fluorescence biodistribution of vital organs (heart, liver, spleen, lung, kidney, and pancreas) at 12, 24, and 48 h post-injection.


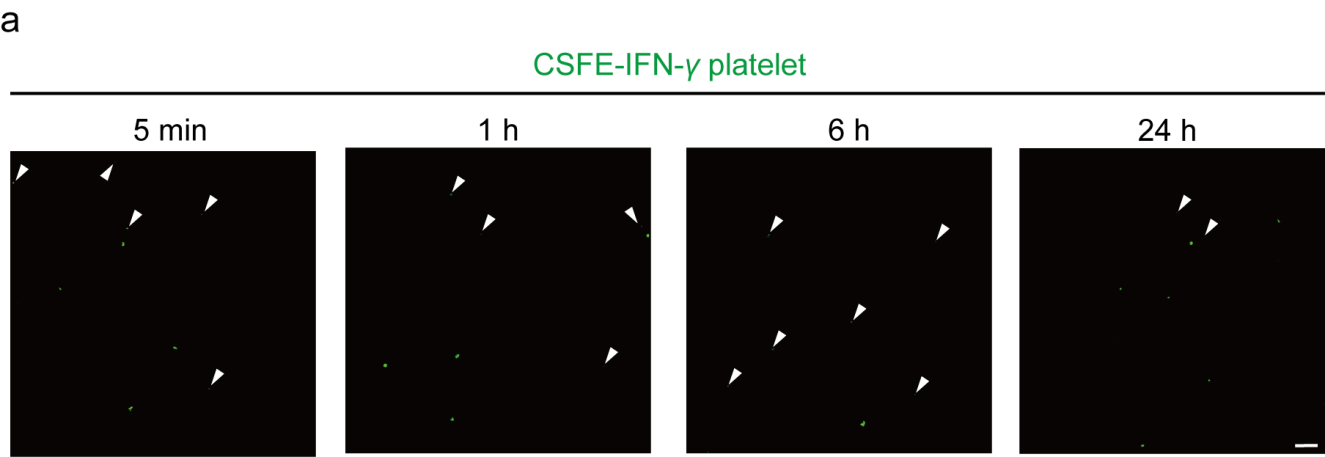


**Figure S15**. The confocal images of the PMPs released from the platelets *in vivo*. The platelets were collected from the blood at different time points after injection into the NOD mice. The white arrows indicate the PMPs. Scale bar: 10 µm.


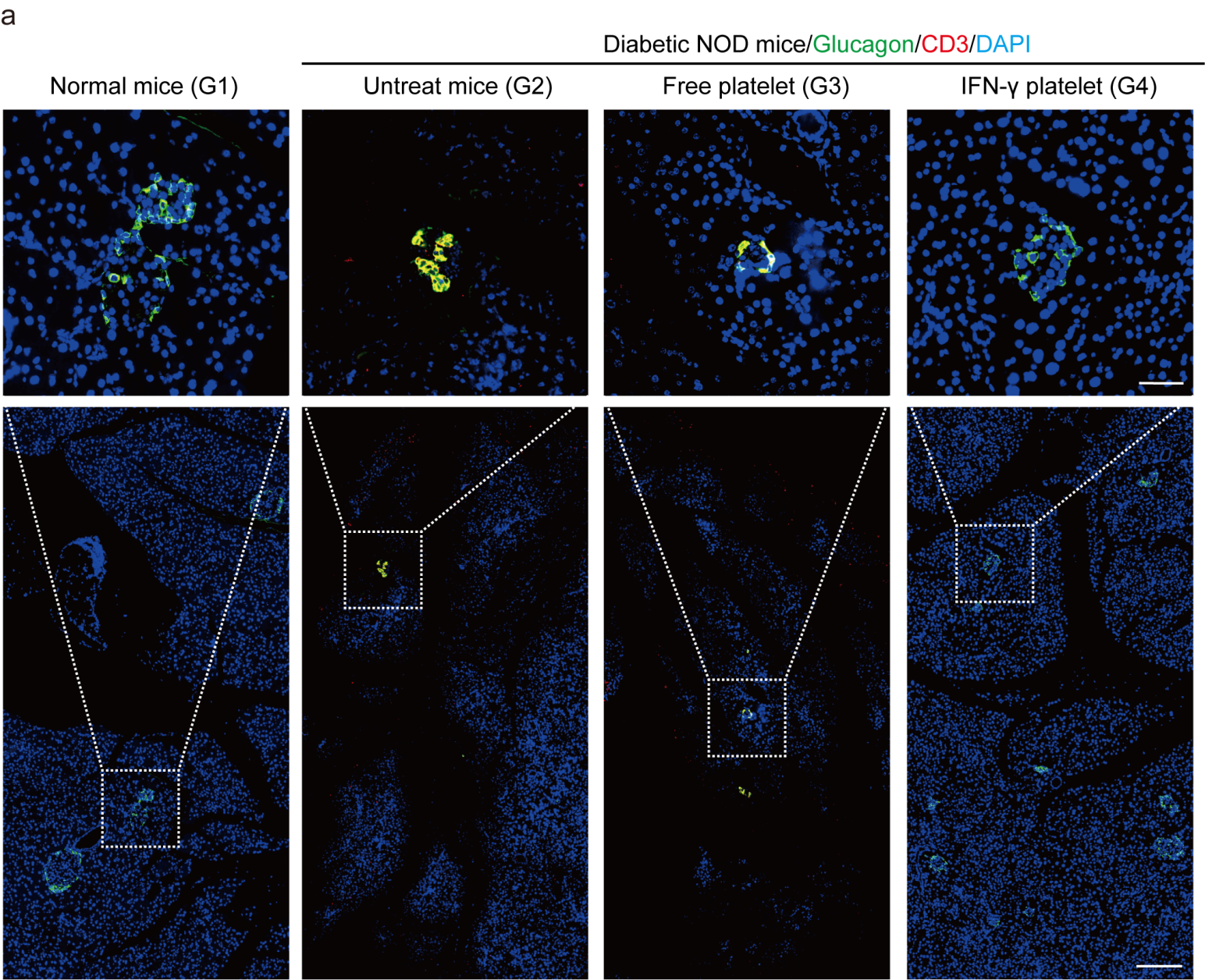


**Figure S16.** Immunofluorescence analysis of CD3^+^ T cells in the pancreas of normal and treated mice. (scale bar: 200 µm in inserts; 50 µm in enlarged images.)


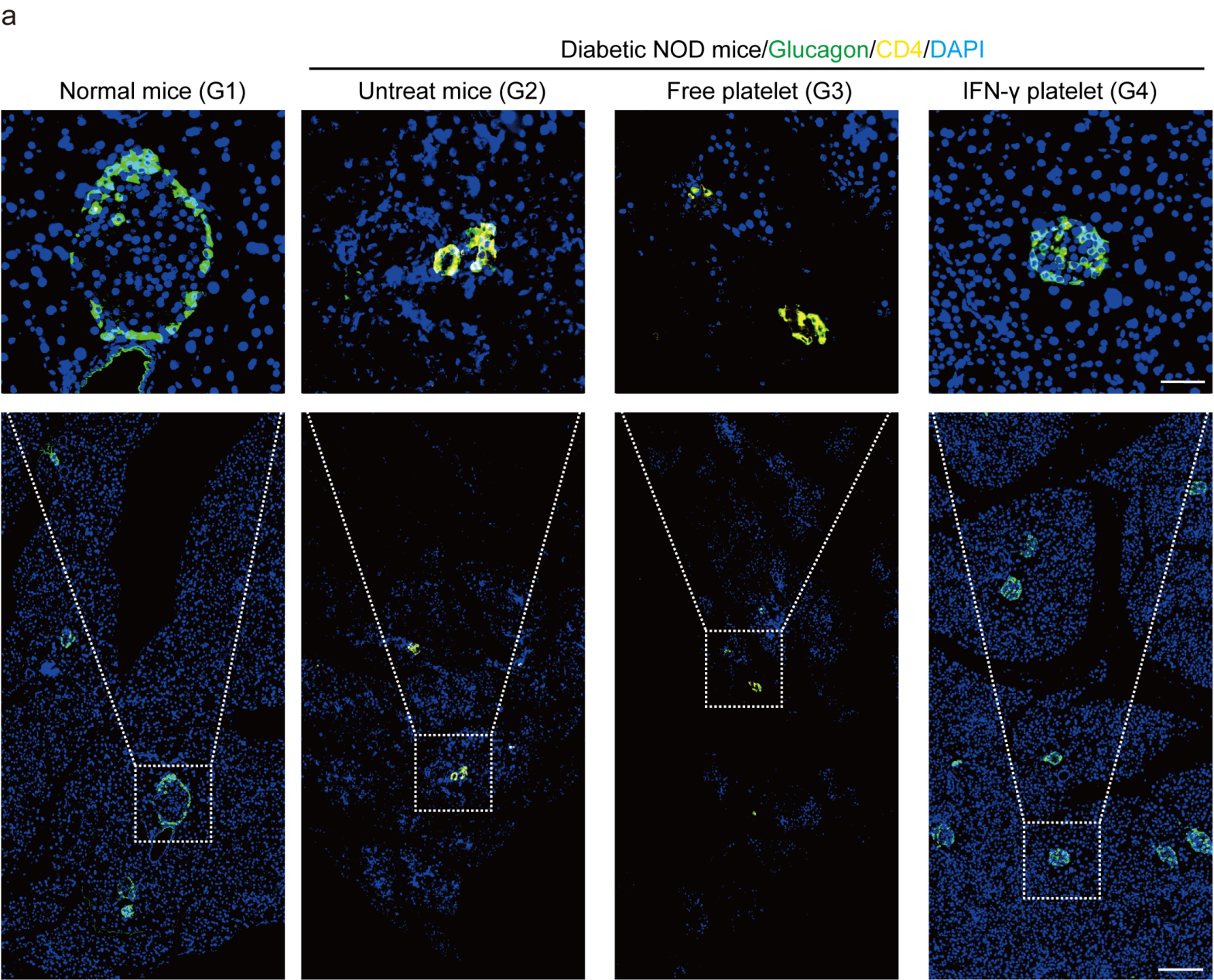


**Figure S17.** Immunofluorescence analysis of CD4^+^ T cells in the pancreas of normal and treated mice. (scale bar: 200 µm in inserts; 50 µm in enlarged images.)


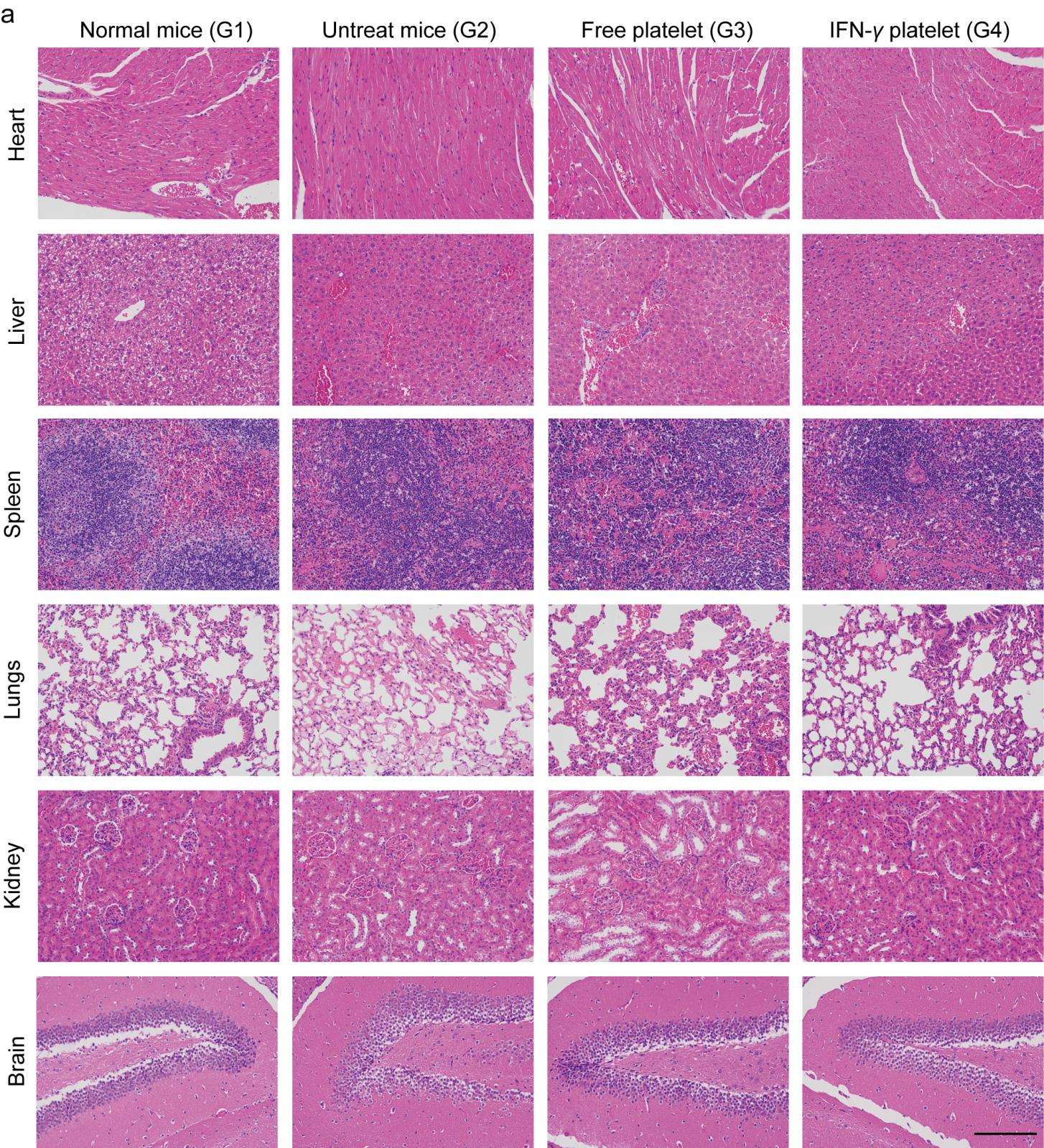


**Figure S18**. Typical H&E staining images of heart, liver, spleen, lung, and kidney slices from healthy mice after different treatments. Scale bar: 50 µm.


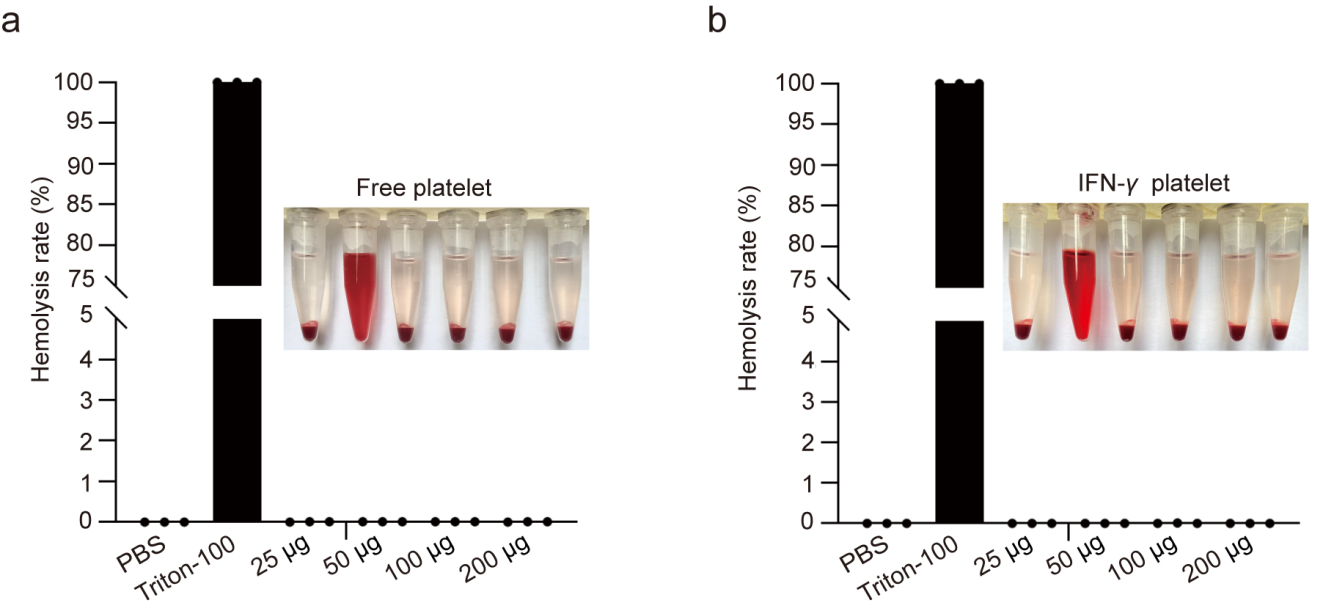


**Figure S19**. Hemolysis rates of Free platelets and IFN-*γ* platelets after incubation with mouse erythrocytes (*n* = 3).
